# Euphosantianane E–G: Three New Premyrsinane Type Diterpenoids from *Euphorbia sanctae-catharinae* with Contribution to Chemotaxonomy

**DOI:** 10.3390/molecules24132412

**Published:** 2019-06-29

**Authors:** Abdelsamed I. Elshamy, Tarik A. Mohamed, Saud L. Al-Rowaily, Ahmed M. Abd-ElGawad, Basharat A. Dar, Abdelaaty A. Shahat, Mohamed-Elamir F. Hegazy

**Affiliations:** 1 Natural Compounds Chemistry Department, National Research Centre, 12622, 33 El Bohouth St., Dokki, Giza 12622, Egypt; 2 Chemistry of Medicinal Plants Department, National Research Centre, 33 El-Bohouth St., Dokki, Giza 12622, Egypt; 3 Plant Production Department, College of Food & Agriculture Sciences, King Saud University, P.O. Box 2460 Riyadh 11451, Saudi Arabia; 4 Department of Botany, Faculty of Science, Mansoura University, Mansoura 35516, Egypt; 5 Department of Pharmacognosy, College of Pharmacy, King Saud University, P.O. Box 2457, Riyadh 11451, Saudi Arabia; 6 Department of Pharmaceutical Biology, Institute of Pharmacy and Biochemistry, University of Mainz, Staudinger Weg 5, 55128 Mainz, Germany

**Keywords:** endemic plant, *Euphorbia sanctae-catharinae*, euphorbiaceae, premyrsinane diterpenoids, euphosantianane E–G, chemotaxonomic significance

## Abstract

*Euphorbia* species were widely used in traditional medicines for the treatment of several diseases. From the aerial parts of Egyptian endemic plant, *Euphorbia sanctae-catharinae*, three new premyrsinane diterpenoids, namely, euphosantianane E–G (**1**–**3**), alongside four known triterpenes, 9,19-cyclolanostane-3β,24*S*-diol (**4**), 25-methoxycycloartane-3β,24*S*-diol (**5**), 25-methylenecycloartan-3β,24*R*-diol (**6**), and 25-methylenecycloartan-3β,24*S*-diol (**7**), were isolated and identified. The chemical structures were proven depending upon spectroscopic analysis, including FTIR, HRFABMS, and 1D/2D-NMR. The chemotaxonomic significance of the isolated compounds, especially diterpenes from *E. sanctae-catharinae* compared to those documented from different *Euphorbia* species was also studied via agglomerative hierarchical clustering (AHC). The Egyptian endemic *Euphorbia sanctae-catharina* was grouped with *E. bupleuroides*, *E. fidjiana*, *E. fischeriana*, *E. pithyusa* subsp. *cupanii*, *E. prolifera*, and *E. seguieriana*, where myrsinol diterpenoids were the characteristic compounds.

## 1. Introduction

From the times of ancient Egyptian civilization, medicinal plants have been used as resources of several medicinal treatments. Egypt has unique biodiversity, including different ecological zones such as the Nile-Delta areas, Mediterranean coasts, Red Sea coast, and deserts. Because of these variations in ecological and natural factors between these zones, it is characterized by a diversity of wild and/or endemic medicinal plants. It also includes 13 pharmacopeia ones, 60 endemic ones, and 529 species used for medical purposes [1,2]. The Sinai Peninsula and especially Saint Katherine is one of the most interesting and promising resources of medicinal plants. The medicinal plants located in Sinai have shown unique phytochemicals with potent biological activities which have piqued the interest of many scientists, especially biologists and phytochemists, for further investigations [1,3,4,5].

It is well known that *Euphorbia* species all over the world have different chemical and biological diversity. Several *Euphorbia* plants have been used in folk medicines against several remedies, such as skin diseases, warts, gonorrhea, migraine, and intestinal parasites. Phytochemical characterization of these plants affords highly bioactive isoprenoids [6,7,8]. Diterpenes represent the main constituents from the plants of this genus, including jatrophanes, premyrsinanes, Abietanes, tiglianes, ingenanes, lathyranes, and myrsinols [6,7,9].

Several bioactivities were described for the different extracts and isolated metabolites of these plants, such as anti-inflammatory [10], antifungal [11], antiviral [12], antispasmodic [11], cytotoxic [9,13], antimutagenic [14], antibacterial [15,16], and hepatoprotective [6,7].

**Figure 1:**
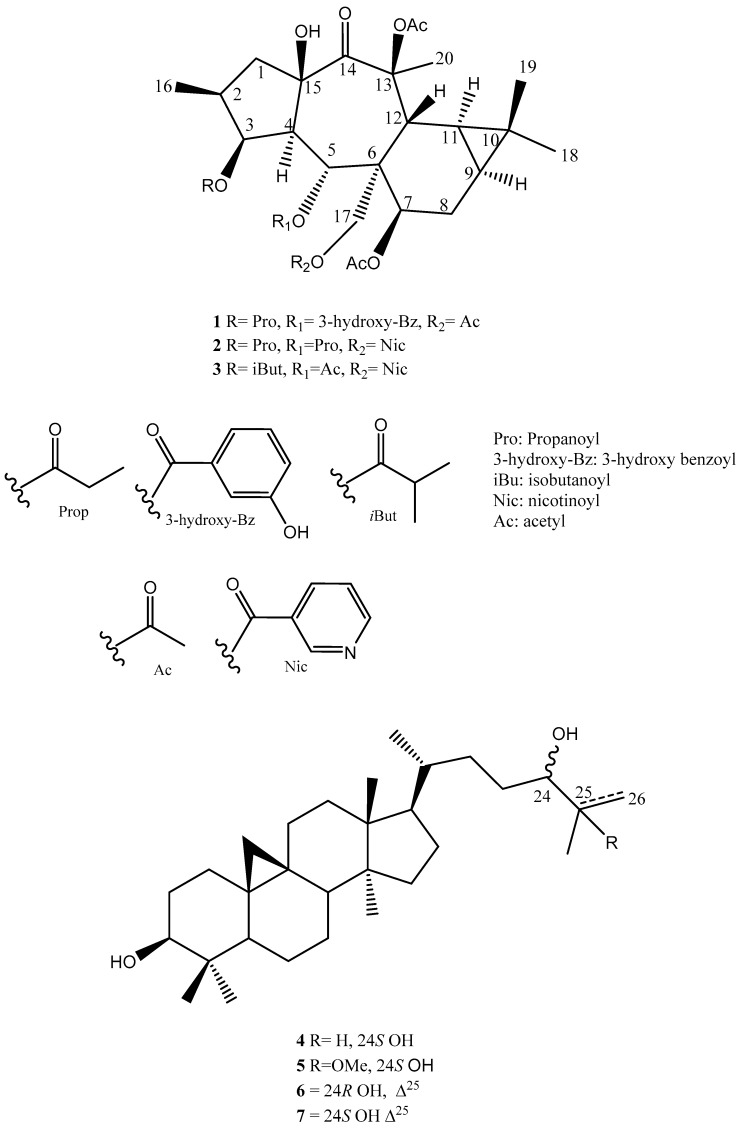
Isolated compounds from *Euphorbia sanctae-catharinae*.

Among the 60 identified endemic species to Egypt, three *Euphorbia* species (*E. bivonae* Steud., *E. punctata* Delile, and *E. sanctae-catharinae* Fayed (synonym of *E. obovata* Decne.)) were recorded. Recently, our group successfully isolated and identified nine premyrsinanes diterpenoids as well as three flavonoids from *E. sanctae-catharinae* and evaluated their cytotoxic activities [9]. Continuing our work on the investigation of new metabolites from medicinal plants, we performed here the isolation and identification of seven terpenoids, including three new premyrsinanes (**1**–**3**, Euphosantianane E–G), alongside four known triterpenes (**4**–**7**) (Figure 1).

## 2. Results and Discussion

### 2.1. Structure Elucidation of the Isolated Compounds

The chemical characterization of the CH_2_Cl_2_–MeOH extract of the air-dried powder of the Egyptian endemic plant, *E. sanctae-catharinae*, using different chromatographic tools, afforded three new premyrsinane types diterpenes (**1**–**3**) alongside four known triterpenes (**4**–**7**), presented in Figure 1. The chemical structures of these seven isolated compounds were established depending upon the modern spectroscopic analysis.

Euphosantianane E (**1**) was isolated as a colorless oil and exhibited positive optical rotation (${\left[\alpha \right]}_{D}^{25}$ + 18.0 in MeOH). The chemical formula was assigned as C_36_H_46_O_13_ (cal. 686.2938) depending upon the HRFABMS that exhibit molecular ion peak at *m/z* 709.2930 (M + Na)^+^ and displayed 14 degrees of unsaturation. FTIR absorption bands characteristic for OH and carbonyl esters were observed, respectively, at 3380 and 1732 cm^−1^, alongside with the aromatic ring absorption bands at 1442 and 723 cm^−1^. ^1^H-NMR spectrum (Table 1) of **1** exhibited proton signals for three oxygenated methines at *δ_H_* 5.35 br t (*J* = 3.48 Hz), 6.42 d (*J* = 11.46 Hz) and 4.77 d (*J* = 6.72 Hz); one methylene at *δ*_H_ 4.35 d (*J* = 11.70 Hz) and 4.67 d (*J* = 11.70 Hz); three characteristic methyles singlets of acetates at 2.11 s (6H) and 2.12 s. Additionally, four singlet methyl signals at *δ_H_* 0.94 s, 1.04 s (6H), and 1.57 s, as well as one triplet methyl signal at *δ_H_* 0.98 t (*J* = 7.5 Hz). Additionally, two methine protons characteristic to cyclopropane moiety were identified at *δ_H_* 0.73 m (2H). ^13^C-NMR (Table 1) spectrum displayed 36 carbon signals that were characterized by DEPT-135 and HMQC experiments to 12 quaternary carbons (including five carbonyl esters at *δ_C_* 173.5, 170.8 (2 × C), and 170.0, 168.9; one ketone at *δ_C_* 204.1 and two oxygenated carbons at *δ_C_* 84.2 and 85.7); 12 methines (including three oxygenated at *δ_C_* 78.2, 70.6 and 70.5); four methylene carbons (including one oxygenated at *δ_C_* 62.8); and eight methyl groups (including three methyles of acetates at *δ_C_* 20.8, and 21.4 (2 × C) and one methyl of propanoyl group at *δ_C_* 8.8). 1D and 2D-NMR spectra of **1** were closely related to previously reported premyrsinanes with clear differences in type of substitution in C-5. A complete assignment of **1** was compatible with previously reported euphosantianane A [9].

**Figure 2:**
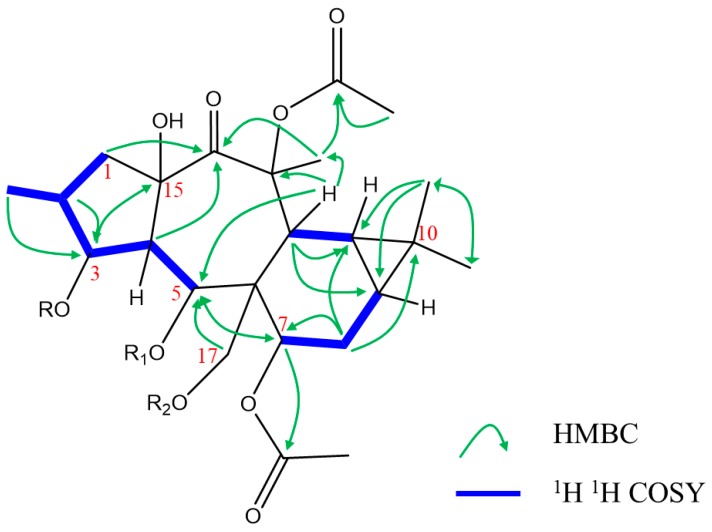
Selected significant ^1^H ^1^H COSY, and Key HMBC correlations of **1**–**3**.

^1^H–^1^H COSY correlations of H-4 (*δ_H_* 2.37, dd, *J* = 3.78, 11.5 Hz)/H-3 (*δ_H_* 5.35 brt, *J* = 3.48 Hz), H-4/H-5 (*δ_H_* 6.42 d, *J* = 11.46 Hz), H-9 (*δ_H_* 0.73 m)/H-8 (*δ_H_* 3.50 brd, *J* = 6.54 Hz), and H-8/H-7 (*δ_H_* 4.77, d, 6.72 Hz) confirmed the oxygenation of C-3 (*δ_C_* 78.2), C-5 (*δ_C_* 70.5), and C-7 (*δ_C_* 70.6). Additionally, H_3_-16 (*δ_H_* 1.04, s), H-12 (*δ_H_* 3.48, s), and H-5 showed correlation with C-3, C-5, and C-7 in HMBC analyses, respectively (Figure 2). The HMBC spectra showed the following correlation: H-17 at *δ_H_* 4.67 (d, *J* = 11.70 Hz)/C-5 (*J*^3^); H-3/C-15 (84.2; *J*^3^); H-4/C-15 (*J*^2^); H-4/C-14 (204.1; *J*^3^); H_3_-20 at *δ_H_* 1.57(s)/C-14 (*J*^3^); H-12/C-13 (*δ_C_* 85.7; *J*^2^) confirmed the hydroxylation of C-15, oxygenation of C-13 and C-17, and localization of ketone group in C-14 (Figure 2). As described in several reports, the premyrsinanes isolated from *Euphorbia* plants are usually characterized by variation of functionality at C-3, C-5, C-7, and/or C-17 [9,17,18]. The 1D and 2D-NMR as well as mass spectrum exhibited the replacement of 3-methylbutyryl moiety (euphosantianane A, Hegazy et al., 2018) with 3-hydroxy benzoyl moiety. This substitution was deduced depending upon the HMBC correlations of H-5/(O=C)-1`` (*δ*_C_ 168.9; *J*^3^), H-3`` at *δ*_H_ 7.39 d (*J* = 1.56 MHz)/C-1``, and H-7`` at *δ*_H_ 7.58 dd (*J* = 1.68, 7.98 MHz). Further, the hydroxylation of C-4`` was confirmed with the aromatic quaternary carbon at *δ_C_* 161.9 (C-4``) alongside the HMBC correlations of H-6`` at *δ*_H_ 6.80 t (*J* = 9.18 MHz)/C-4``.

**Figure 3:**
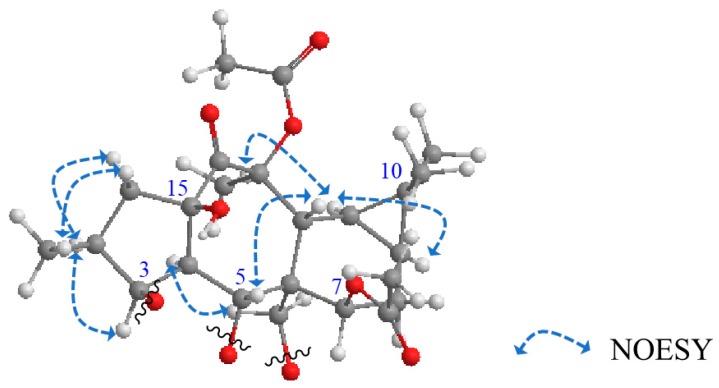
Significant NOESY correlations of **1**–**3**.

The relative configuration of **1** was determined depending upon NOESY correlations (Figure 3). The *trans* and *α* orientation H-4 and H_2_-17, respectively, was established depending upon the biosynthetic pathways of these types of myrsinols [9,17,18]. Starting from this reference point, NOESY correlations of H-4*α*/H-3 and H-3/H-2 deduced the *α* configuration of H-3 and H-2. NOESY correlations of H-12*β*/H-5, H-9*α*/H-11*α*, and H-11*α*/H-20 elucidated the *β* orientation of H-5 and Me-20. From all these described data, **1** was assigned as premyrsinol-3*β*-propanoyl-5*α*-(3-hydroxy)-benzoyl-7*β*,13*β*,17*α*-triacetate (Euphosantianane E).

Euphosantianane F (**2**, Figure 1) was isolated as a colorless oil and exhibited positive optical rotation (${\left[\alpha \right]}_{D}^{25}$ + 24.4 in MeOH). Based on the HRFABMS ion peak at *m/z* 685.3089 (M), the molecular formula of **2** was determined as C_36_H_47_NO_12_ (cal. 685.3098), exhibiting 14 degrees of unsaturation. FTIR bands for OH and carbonyl esters were characterized at 3441 and 1736 cm^−1^, respectively, in addition to the aromatic ring absorption bands at 1434 and 741 cm^−1^. 1D-NMR data (Table 1) and 2D (^1^H–^1^H COSY, HMQC, and HMBC, Figure 2) exhibited that **2** is very close to euphosantianane A (Hegazy et al., 2018), except the substitution at C-5 and C-17 was replaced by propanoyl and nicotinoyl, respectively. This substitution was deduced by the downfield shift of C-5 by 1.9 ppm to be at *δ_C_* 70.7 and the upfield shift of C-17 by 1.0 ppm to be at *δ_C_* 62.6, in addition to the HMBC and ^1^H–^1^H COSY related to propanoyl and nicotinoyl substituents, respectively. Depending on NOESY, compound **2** showed the same stereochemistry of **1**. Thus, **2** was identified as premyrsinol-3*β*,5*α*-dipropanoyl-7*β*,13*β*-diacetyl-17*α*-nicotinoate (euphosantianane F).

Euphosantianane G (**3**, Figure 1) was obtained as a colourless oil and exhibited positive optical rotation (${\left[\alpha \right]}_{D}^{25}$ 53.2 in MeOH). The HRFABMS ion peak assigned at *m/z* 708.3090 (M + Na)^+^ indicated the molecular formula as C_36_H_47_NO_12_ (cal. 685.3098) with 14 degrees of unsaturation. Hydroxyls and carbonyl ester FTIR bands at 3430 and 1728 cm^−1^, respectively, as well as the aromatic ring absorption bands at 1458 and 716 cm^−1^ were identified. 1D-NMR data (Table 1), ^1^H–^1^H COSY and HMBC (Figure 2) of **3** are very close to euphosantianane D [9] (Hegazy et al., 2018), with the usual exception of different substitutions at C-3 and C-5, in which isobutanoyl and acetyl groups were inserted, respectively. The downfield shift of C-3 by 1.3 ppm found at *δ_C_* 78.6 deduced the substitution of this carbon with the acetyl group instead of 3-dimethylbutanoyl in euphosantianane D [9]. One methylene group was detected in ^1^H and ^13^C-NMR at *δ*_H_ 2.15 m and *δ*_C_ 27.6, respectively, along with at two methyle groups at *δ*_H_ 1.05 d (*J* = 7.02 Hz; 6H) and at *δ_C_* 8.9 that were characterized by DEPT-135 and HMQC. Strong ^1^H–^1^H COSY and HMBC correlations of H_3_-3`/H-2`, H_3_-3`/C-2` (*J*^2^), H_3_-3`/(C=O)-1` (*δ*_C_ 175.1, *J*^3^), and H-3 at *δ*_H_ 5.20 s/(C=O)-1` were observed that deduced the localization of isobutanoyl group at C-3 (*δ*_C_ 78.6). The NOESY experiment confirmed that **3** has the same absolute configuration of **1** and **2**. Thus, **3** was identified as premyrsinol-3*β*-isobutyroyl-5*α*,7*β*,13*β*-triacetoyl-17*α*-nicotinoate (euphosantianane G, **3**).

In addition to these three new premyrsinane diterpenes, euphosantianane E–G (**1**–**3**), four known triterpenes, 9,19-cyclolanostane-3β,24*S*-diol (**4**) [19], 25-methoxycycloartane-3β,24*S*-diol (**5**) [20], 25-methylenecycloartan-3β,24*R*-diol (**6**), and 25-methylenecycloartan-3β,24*S*-diol (**7**) [21,22], were characterized (Figure 1).

### 2.2. Study of Chemosystematic Significance

*Euphorbia* is one of the most diverse and very large genera among flowering plants. It has a worldwide distribution, and it is found as herbs, shrubs or trees. It is characterized by the presence of milky latex. More than 2000 species are distributed at cosmopolitan, but especially tropical, subtropical, and warm-temperate regions [23]. *Euphorbia* is represented by 36 species in the flora of Egypt. Three of these *Euphorbia* are endemic to Egypt, namely, *E. bivonae* Steud., *E. punctata* Delile, and *E. sanctae-catharinae* Fayed (synonym of *Euphorbia obovata* Decne).

In our previous work on the chemical composition of the endemic *E. sanctae-catharinae* of Egypt, nine premyrsinanes diterpenes and three flavonoids were isolated and identified from *E. sanctae-catharina*. Moreover, in the present study, four cycloartane triterpenes and three new myrsinol compounds were identified for the first time from the endemic *E. sanctae-catharinae*.

**Figure 4:**
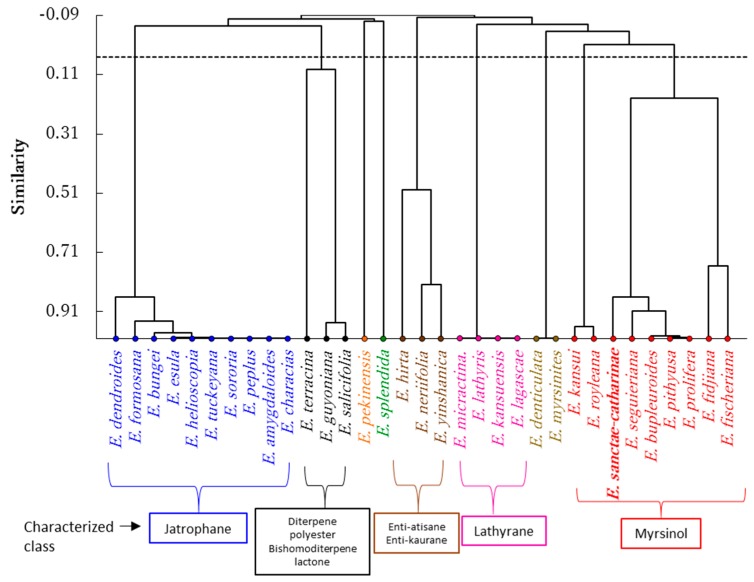
Agglomerative hierarchical clustering (AHC) of 32 *Euphorbia* species and the present studied endemic *Euphorbia* (*E. sanctae-catharinae*) based on the terpenoid compounds.

In order to correlate the chemical composition of this endemic species to Egypt with other *Euphorbia* species, agglomerative hierarchical clustering (AHC) analysis was performed. Based on diterpenoid chemical composition from 32 *Euphorbia* species in addition to *E. sanctae-catharinae*, the AHC analysis revealed these plants categorized into nine groups (Figure 4). The largest group comprised ten *Euphorbia* species (*E. amygdaloides*, *E. bungei*, *E. characias*, *E. dendroides*, *E. esula*, *E. formosana*, *E. helioscopia*, *E. peplus*, *E. sororia*, and *E. tuckeyana*). These species showed a close correlation to each other due to the presence of jatrophane diterpenoid compounds. These species mainly contained jatrophane terpenoids, where amygdaloidins A–L were reported in *E. amygdaloides* [24]. Helioscopinolides A, B, and C were reported in *E. formosana* [25], while euphocharacins A–L were isolated from *E. characias* [26]. The jatrophane diterpenoids euphodendrophanes A–P, abeodendroidin, and epiabeodendroidin F were isolated from *E. dendroides* [27,28,29], while esulatin A–M, esulone A, and esulone B were isolated from *E. esula* [30].

Corea et al. [31] identified several types of jatrophane compounds in *E. peplus*, such as pepluanin A, B, and C, euphopeplin A, 2*α*,5*α*,*7β*,8*α*,9*α*, 14*β*-hexaacetoxy-3*β*-benzoyloxy-15-hydroxyjatropha-6(17), 11*E*-diene, and 5*α*,8*α*,9*β*,10*β*, 14*α*-pentaacetoxy-3*β*-benzoy-loxy-15-hydroxypepluane. However, *E. helioscopia* was reported as a rich plant with jatrophane diterpenes, where it contained euphohelin A–E, euphoheliosnoids A–C, and helioscopianoids A–Q [32,33,34]. Sororianolide A–C were identified from *E. sororia* [35], while tuckeyanol A and B and euphotuckeyanol were isolated from *E. tuckeyana* [36].

Therefore, the predominance of jatrophane compounds in theses *Euphorbia* species reflected the similarity of the chemistry of these species and led to a clustering of them together in one group of the AHC (Figure 4). Nevertheless, different diterpenoid classes such as lathyrane, *enti*-atisane, ingole, and bishomoditerpene lactone were also isolated from some of these species [24,34].

On the other hand, *E. kansuensis*, *E. lagascae*, *E. lathyris*, and *E. micractina* were closely related to each other and grouped together (Figure 4). This group was characterized by lathyrane-type diterpenoids, where *E. lagascae* has been reported to possess latilagascene A–F and jolkinol B [37]. While euphorbia factor L_8_, euphorbia factor L_7a_ and _7b_, euphorbia factor L_3_, jolkinol B, isolathyrol, 7-hydroxylathyrol, and lathyrol were isolated from *E. lathyris* [38,39].

Another group comprised *E. hirta*, *E. neriifolia*, and *E. yinshanica* and was characterized by the presence of *enti*-atisane and *enti*-kaurane diterpenoids [40,41,42]. *Euphorbia guyoniana*, *E. salicifolia*, and *E. terracina* represented another group, and these plants are characterized by the presence of diterpene polyester and bishomoditerpene lactone. Terracinolide A and B were identefied in *E. terracina* [43], guyonianin C–F were isolated from *E. guyoniana* [44,45], and euphosalicin 1–3 as well as salicinolide were idenfied in *E. salicifolia* [46].

Our studied endemic species, *E. sanctae-catharinae*, was grouped with *E. bupleuroides*, *E. fidjiana*, *E. fischeriana*, *E. pithyusa* subsp. *cupanii*, *E. prolifera*, and *E. seguieriana*. However, the Pearson correlation coefficient analysis revealed that *E. sanctae-catharinae* showed a close correlation to *E. bupleuroides* (0.888), followed by *E. prolifera* (0.880), then *E. pithyusa* subsp. *cupanii* (0.870) based on the composition of the terpenoid composition (Figure 4). Myrsinol diterpenoids were the main constituents of these *Euphorbia* species [9,47,48,49], while other diterpenoid compounds such as abietane, *ent*-kaurane, *ent*-atisane, and lathyrane types as well as cycloartane triterpene were also identified in these plants [6,50]. Hegazy et al. [9] identified several myrsinol compounds form *E. sanctae-catharinae*, such as euphosantianane A–D. In addition, the present study revealed the presence of an additional three new myrsinol compounds (euphosantianane E–G). However, *E. prolifera* was richer in the myrsinol diterpenoids where it comprises numerous compounds including for examples, proliferin A–D, euphorprolitherin B and D, euphorbiaproliferin A–I, and euphorbialoid [17,47,48,49,51,52].

*Euphorbia pithyusa* subsp. *cupanii* was reported to have myrsinol compounds [18], while Jeske et al. [50] stated that *E. seguieriana* contained 12 myrsinol compounds. Therefore, the results exhibited that our studied endemic species (*E. sanctae-catharinae*) was grouped with theses *Euphorbia* species due to the predominance of myrsinol diterpenes. Nevertheless, the present phytochemical study showed the presence of four cycloartane triterpenes (25-methylenecycloartan-3β,24*R*-diol, 9,19-cyclolanostane-3β,24*S*-diol, and 25-methylenecycloartan-3β,24*S*-diol,) which was already reported from some *Euphorbia* species like, *E myrsinites* [53], *E. denticulate* [54], and *E. spinidens* [55].

On the other hand, cycloartane triterpenes were identified in both *E. denticulate* and *E. myrsinites*, and thus grouped together. However, *E. kansui* and *E. royleana* were similar to each other, chacterized by ingole compounds [56,57,58], while either *E. pekinensis* or *E. splendida* showed dissimilarity with all tested *Euphorbia* species.

From previously reported flavonoids from *Euphorbia* species, flavonoid and their glycosides were isolated and identified from some of these plants, such as *E. condylocarpa*, *E. virgata*, *E. chamaesyce*, *E. hirta*, and *E. magalanta* [59,60,61,62], in addition to *E. sanctae-catharinae* [9]. For example, quercetin-3-*O*-*α*-rhamnopyranoside and kaempferol-3-*O*-rhamnoside were isolated from *E. condylocarpa* collected from Iraq [60], and this is in total agreement with the isolated compound from *E. sanctae-catharinae*.

## 3. Conclusions

From the Egyptian endemic plant, *E. sanctae-catharinae*, three new premyrsinanes, euphosantianane E–G (**1**–**3**), alongside the known triterpenes (**4**–**7**), were characterized using modern spectroscopic tools. For first time, the chemotaxonomic significance of isolated compounds from *E. sanctae-catharinae* especially diterpenes compared to those documented from different *Euphorbia* ecospecies was also studied, and it was found to be closely correlated to *E. bupleuroides*, *E. prolifera*, and *E. pithyusa* subsp. *cupanii* based on the composition of the terpenoid compound classes.

## 4. Materials and Methods

### 4.1. General Experimental Procedures

As described in our previously reported protocol by Hegazy et al. [9].

### 4.2. Plant Material

The aerial parts of *E. sanctae-catharinae* were collected from Wadi Jibaal, Protectorate of Saint Katherine, South Sinai, Egypt during the flowering stage in April 2016 under the permission of the Protectorate for scientific purposes. A voucher specimen (#16-212) has been deposited in the herbarium of the National Research Centre. The authentication of the plant was kindly performed by Prof. Mona Marzouk, Professor of Taxonomy, NRC, Cairo, Egypt.

### 4.3. Extraction and Isolation

Air dried aerial parts (one kg) were ground, and extracted with CH_2_Cl_2_:MeOH (1:1) at room temperature, filtered and then concentrated under vacuum afforded black gum (63 g). The extract was then subjected to silica gel flash column chromatography (5 × 60 cm) and eluted with n-hexane/EtOAc step gradient. Eight main fractions (ES-1:ES-8) were obtained after thin layer chromatography (TLC, Kieselgel 60 F254, 0.25 mm, Merck, Darmstadt, Germany) examinations of similar ones. Fraction ES-4 (724 mg), subjected to further fractionation via reversed phas ODS column (3×60 cm) with MeOH: H_2_O 4:1, afforded 3 main subfractions (ES-4A–C) after TLC examinations. Subfraction ES-4B, eluted by MeOH:H_2_O (4:1) by a reversed phase HPLC (20 × 250 cm), afforded compounds **1** (2.8 mg), **3** (3.2 mg), **6** (14.6 mg), and **7** (12.9 mg). Moreover, subfraction ES-5 (564 mg) was further fractionated over ODS column (3 × 60 cm) with MeOH: H_2_O 7:3 that afforded 2 main subfractions (ES-5A,B). Subfraction ES-5B was eluted by MeOH:H_2_O (85:15) over a reversed phase HPLC (20 × 250 cm) and afforded compounds **2** (3.4 mg), **4** (15.1 mg), and **5** (7.8 mg).

### 4.4. Spectroscopic Data of Euphosantianane E–G (***1***–***3***)

Euphosantianane E (premyrsinol-3β-propanoyl-5α-(3-hydroxy)-benzoyl-7β,13β,17α-triacetate, **1**): colorless oil; ${\left[\alpha \right]}_{D}^{25}$ +18.0 (c 0.01, MeOH); FT-IR (KBr): 3380, 1732, 1462 and 728 cm^−1^; ^1^H and ^13^C-NMR spectral data, see Table 1 and Supplementary Materials (S1–S9); HRFABMS: *m/z* 709.2930 (M + Na)^+^; C_36_H_46_O_13_ (cal. 686.2938).

Euphosantianane F (premyrsinol-3β,5α-dipropanoyl-7β,13β-diacetyl-17α-nicotinoate, **2**): colorless oil; ${\left[\alpha \right]}_{D}^{25}$ + 24.4 (c 0.01, MeOH); FT-IR (KBr): 3441, 1736, 1434 and 741 cm^−1^; ^1^H and ^13^C-NMR spectral data, see Table 1 and Supplementary Materials (S10–S17); HRFABMS: *m/z* 685.3089 (M), C_36_H_47_NO_12_ (cal. 685.3098).

Euphosantianane G (premyrsinol-3β-isobutyroyl-5α,7β,13β-triacetoyl-17α-nicotinoate, **3**): colorless oil; ${\left[\alpha \right]}_{D}^{25}$ + 53.2 (c 0.01, MeOH); FT-IR (KBr): 3430, 1728, 1458 and 716 cm^−1 1^H and ^13^C-NMR spectral data, see Table 1 and Supplementary Materials (S18–S25); HRFABMS: *m/z* 708.3090 (M + Na)^+^, C_36_H_47_NO_12_ (cal. 685.3098).

### 4.5. Statistical Analysis

A data matrix of 15 terpene classes from 33 *Euphorbia* species was subjected to agglomerative hierarchical clustering (AHC). This matrix was designed based on the numbers of the identified terpenoid compounds from 32 *Euphorbia* species (collected from the literature review) as well as that identified in the present study from the endemic species to Egypt (*E. sanctae-catharinae*). This analysis was performed by XLSTAT statistical computer software package, version 2018 (Addinsoft, New York, NY, USA).

## Figures and Tables

**Table 1:** ^1^H (600 Hz) and ^13^C (150 Hz) NMR (CDCl_3_) of **1**–**3**^a,b^.

No.	Euphosantianane E (1)		Euphosantianane F (2)			Euphosantianane G (3)		
	δ_H_	δ_C_	δ_H_		δ_C_	δ_H_	δ_C_	
1*α*	3.15 dd (8.05, 13.86)	42.8, t	2.64 dd (10.85, 14.64)		41.1, t	3.16 dd (8.10, 13.62)	42.8, t	
1*β*	1.64 t (13.26)		1.60 m			1.61 m		
2	2.26 qd (1.56, 7.56)	35.1, d	2.31 m		33.8, d	2.25 m	34.0, d	
3	5.35 br t (3.48)	78.2, d	5.46 br dd (3.06, 6.12)		78.7, d	5.20 s	78.6, d	
4	2.37 dd (3.78, 11.5)	50.1, d	3.01 dd (3.30, 10.50)		50.4, d	2.37 brd (11.6)	50.4, d	
5	6.42 d (11.46)	70.5, d	5.79 d (8.80)		68.5, d	6.23 d (11.52)	69.0, d	
6	-----	47.8, s	-----		48.2, s	-----	47.7, s	
7	4.77 d (6.72)	70.6, d	4.90 d (6.54)		68.8, d	4.68 d (6.54)	70.7, d	
8*α*	3.50 br d (6.54)	22.1, t	2.34 d (7.44)		23.2, t	2.32 d (7.14)	22.4, d	
8*β*	1.84 t (16.92)		1.72 t (13.92)			1.88 br d (17.40)		
9	0.73 m	18.9, d	0.75 m		19.4, d	0.79 m	19.0, d	
10	-----	18.4, s	-----		19.1, s	-----	18.4, s	
11	0.73 m	23.8, d	0.75 m		23.3, d	0.79 m	23.9, d	
12	3.48 s	37.4, d	3.47 br d (6.11)		36.0, d	3.45 br d (3.42)	35.0, d	
13	-----	85.7, s	-----		79.8, s	-----	85.5, s	
14	-----	204.1, s	-----		210.2, s	-----	204.2, s	
15	-----	84.2, s	-----		83.6, s	-----	84.1, s	
16	1.04 s	13.9, q	0.92 d (5.01)		15.2, q	0.77 d (5.04)	14.2, q	
17*α*	4.35 d (11.70)	62.8, t	4.55 d (12.18)		62.6, t	4.49 d (11.82)	64.4, t	
17*β*	4.67 d (11.70)		5.28 d (12.30)			5.86 d (11.94)		
18	0.94 s	29.5, q	1.07 s		28.8, q	1.05 s	29.6, q	
19	1.04 s	14.9, q	0.94 s		15.3, q	0.92 s	14.9, q	
20	1.57 s	25.0, q	1.57 s		23.0, q	1.72 s	24.6, q	
**3-*O*-Prop**			**3-*O*-Prop**			**3-*O*-*i*But**		
CO	-----	173.5, s	CO	-----	172.9	CO	-----	175.1, s
2`	2.26 qd (7.62)	27.5, t	2`	2.32 q (7.14)	27.6, t	2`	2.15 m	27.6, d
3`	0.98 t (7.5)	8.8, q	3`	1.15 t (6.66)	8.8, q	3`	1.05 d (7.02)	8.9, q
**5-*O*-3-hydroxy-Bz**			**5-*O*-Prop**			4`	1.05 d (7.02)	8.9, q
CO	-----	168.9, s	CO	-----	170.1, s	**5-*O*-Ac**		
2``	-----	129.6, s	2``	2.36 q (7.14)	27.6, t	CO	-----	170.1, s
3``	7.39 d (1.56)	111.9, d	3``	0.95 t (7.56)	8.8, q	2``	2.00 s	21.3
4``	-----	161.9, s	**7-*O*-Ac**			**7-*O*-Ac**		
5``	6.92 (8.40)	117.9, d	CO	-----	175.1, s	CO	-----	170.8, s
6``	6.80 t (9.18)	136.1, d	2```	1.94 s	21.5, q	2```	2.10 s	21.4
7``	7.58 dd (1.68, 7.98)	119.2, d	**17-*O*-Nic**			**17-*O*-Nic**		
**7-*O*-Ac**			CO	-----	164.9, s	CO	-----	165.7, s
CO	-----	170.8, s	2````	-----	129.7, s	2````	-----	129.7, s
2```	2.11 s	21.4, q	3````	9.22 s	128.4, d	3````	9.15 s	128.3, d
**13-*O*-Ac**				8.30 d (7.44)	137.4, d	4````	8.21 d (7.50)	137.1, d
CO	-----	170.8, s		7.37 m	123.9, d	5````	7.48 m	123.9, d
2````	2.11 s	21.4, q		7.67 d (7.74)	153.7, d	6````	7.68 d (7.44)	150.4, d
**17-*O*-Ac**			4````					
CO	-----	170.0, s	5````					
2`````	2.12 s	20.8, q	6````					

^a^ Coupling constant [*J*] in Hz are given in parentheses, ^b^ s, quaternary, d, methine, t, methylene, q, methyl.

## Supplementary Materials

The Supplementary Materials are available online.
