# Supplementary material for: Euphosantianane E–G: Three New Premyrsinane Type Diterpenoids from Euphorbia sanctae-catharinae with Contribution to Chemotaxonomy
Source: Molecules. 2019 Jun 29;24(13):2412. doi: 10.3390/molecules24132412 (PMC6651875; doi:10.3390/molecules24132412)
Supplement: Supplementary file 1 [file molecules-24-02412-s001.pdf]

Supplementary material

Euphosantianane E–G: Three new premyrsinane type diterpenoids from *Euphorbia sanctae-catharinae* with contribution to chemotaxonomy

Abdelsamed I. Elshamy[1], Tarik A. Mohamed[2], Saud L. Al-Rowaily[3], Ahmed M. Abd-ElGawad[3,4], Basharat A. Dar[3], Abdelaaty A. Shahat[5], Mohamed-Elamir F. Hegazy[2,6,*]

[1] Natural Compounds Chemistry Department, National Research Centre, 12622, 33 El Bohouth St., Dokki, Giza, Egypt; elshamynrc@yahoo.com.
[2] Chemistry of Medicinal Plants Department, National Research Centre, 33 El-Bohouth St., Dokki, Giza 12622, Egypt; tarik.nrc83@yahoo.com
[3] Plant Production Department, College of Food & Agriculture Sciences, King Saud University, P.O. Box 2460 Riyadh 11451, Saudi Arabia; aibrahim2@ksu.edu.sa; srowaily@ksu.edu.sa
[4] Department of Botany, Faculty of Science, Mansoura University, Mansoura 35516, Egypt; dgawad84@mans.edu.eg
[5] Department of Pharmacognosy, College of Pharmacy, King Saud University, P.O. Box 2457, Riyadh 11451, Saudi Arabia; ashahat@ksu.edu.sa
[6] Department of Pharmaceutical Biology, Institute of Pharmacy and Biochemistry, University of Mainz, Staudinger Weg 5, 55128 Mainz, Germany; mohegazyphyma@hotmail.com; mohegazy@uni-mainz.de

* Correspondence: mohegazyphyma@hotmail; aibrahim2@ksu.edu.sa; Tel.: +49(0)6131-39 25751

	Supplementary material	Page
S1:	FAB-MS of **1**	3
S2:	FAB-MS of **1**	4
S3:	HR-FAB-MS of **1**	4
S4:	^{1}H NMR (600 Hz, $CDCl_3$) of **1**	5
S5:	^{13}C NMR (600 Hz, $CDCl_3$) of **1**	6
S6:	HMQC ($CDCl_3$) of **1**	7
S7:	HMBC ($CDCl_3$) of **1**	8
S8:	^{1}H ^{1}H COSY ($CDCl_3$) of **1**	9
S9:	NOESY ($CDCl_3$) of **1**	10
S10:	FAB-MS of **2**	11
S11:	HR-FAB-MS of **2**	11
S12:	^{1}H NMR (600 Hz, $CDCl_3$) of **2**	12
S13:	^{13}C NMR (600 Hz, $CDCl_3$) of **2**	13
S14:	HMQC ($CDCl_3$) of **2**	14
S15:	HMBC ($CDCl_3$) of **2**	15
S16:	^{1}H ^{1}H COSY ($CDCl_3$) of **2**	16
S17:	NOESY ($CDCl_3$) of **2**	17
S18:	FAB-MS of **3**	18
S19:	HR-FAB-MS of **3**	18
S20:	^{1}H NMR (600 Hz, $CDCl_3$) of **3**	19
S21:	^{13}C NMR (600 Hz, $CDCl_3$) of **3**	20
S22:	HMQC ($CDCl_3$) of **3**	21
S23:	HMBC ($CDCl_3$) of **3**	22
S24:	^{1}H ^{1}H COSY ($CDCl_3$) of **3**	23
S25:	NOESY ($CDCl_3$) of **3**	24

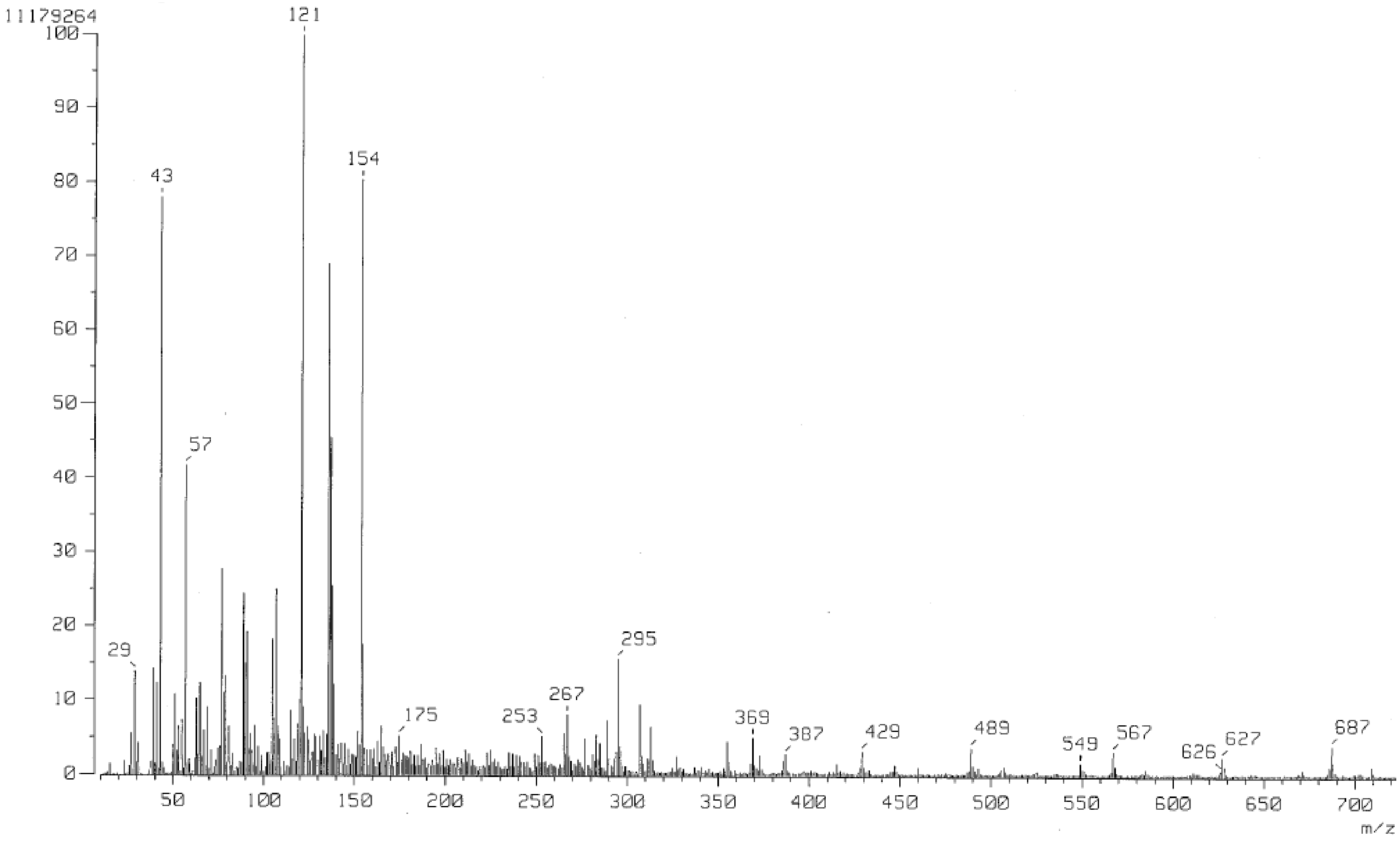

11179264
100
90
80
70
60
50
40
30
20
10
0
121
154
43
57
29
175
253
267
295
369
387
429
489
549
567
626
627
687
50
100
150
200
250
300
350
400
450
500
550
600
650
700
m/z

S1: FAB-MS of **1**

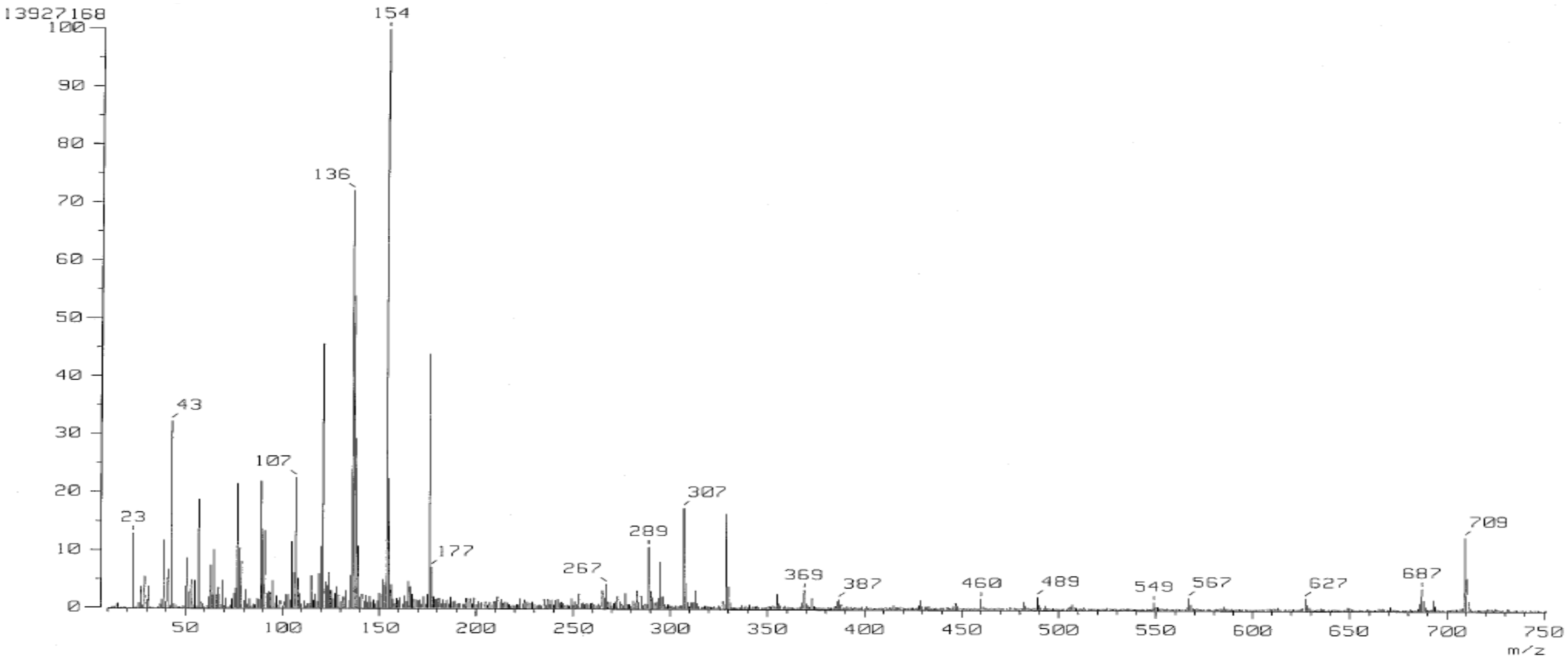

S2: FAB-MS of **1**

Date : 16-Jul-2018 14:42
Instrument: MS700D
Sample: ESS-12
Note: MStation
Inlet: Direct   Ion Mode: FAB+
RT: 4.16 min   Scan#: 19
Elements: C 150/0, H 250/0, O 50/0
Mass Tolerance: 5mmu
Unsaturation (U.S.): 0.0 - 15.0

	Observed m/z	Int %	Err. [ppm / mmu]	U.S.	Composition
1	709.2930	100.00	-1.1 / -0.6	13.5	C36 H46 O13 Na

S3: HR-FAB-MS of **1**

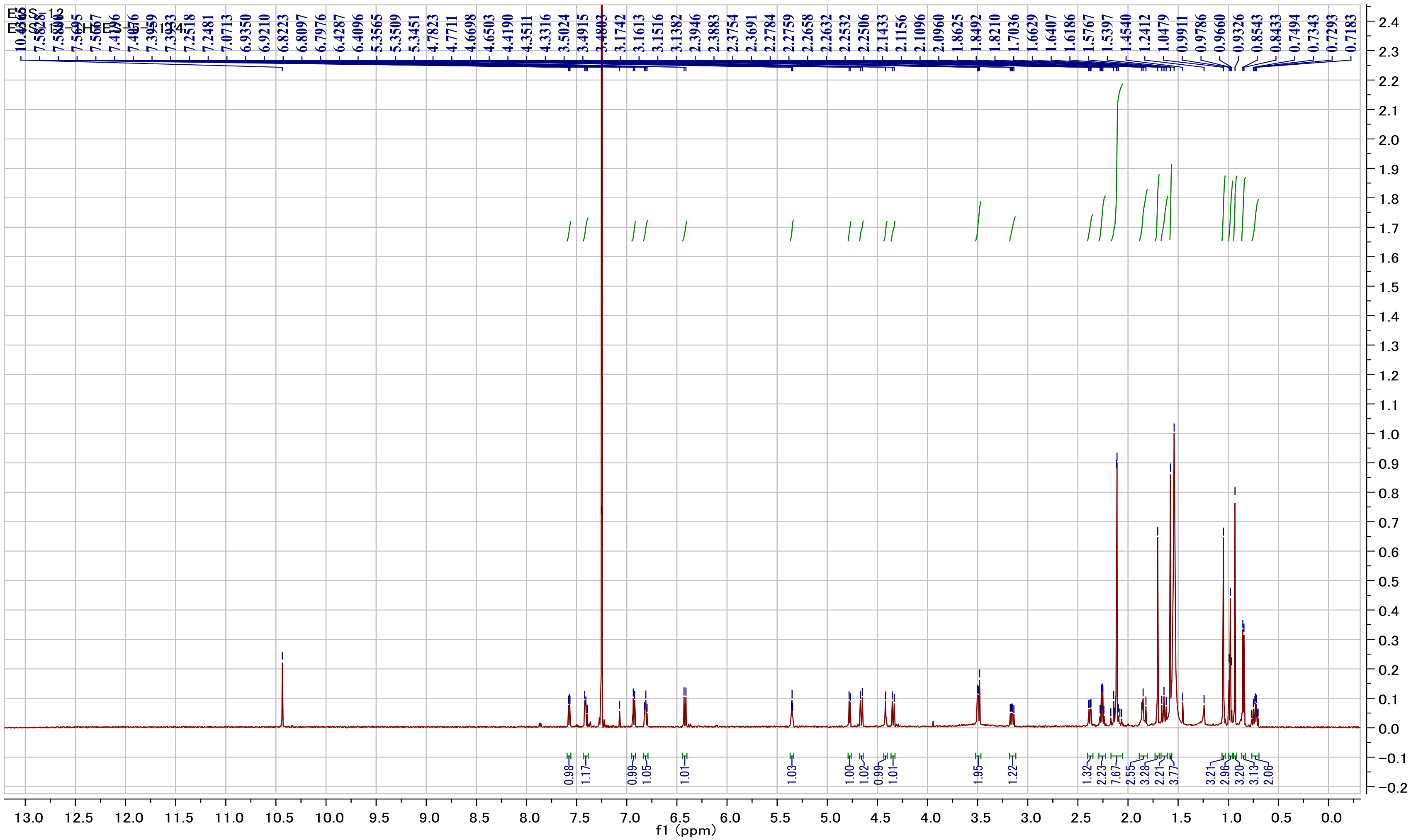

S4: ^{1}H NMR (600 Hz, $CDCl_3$) of **1**

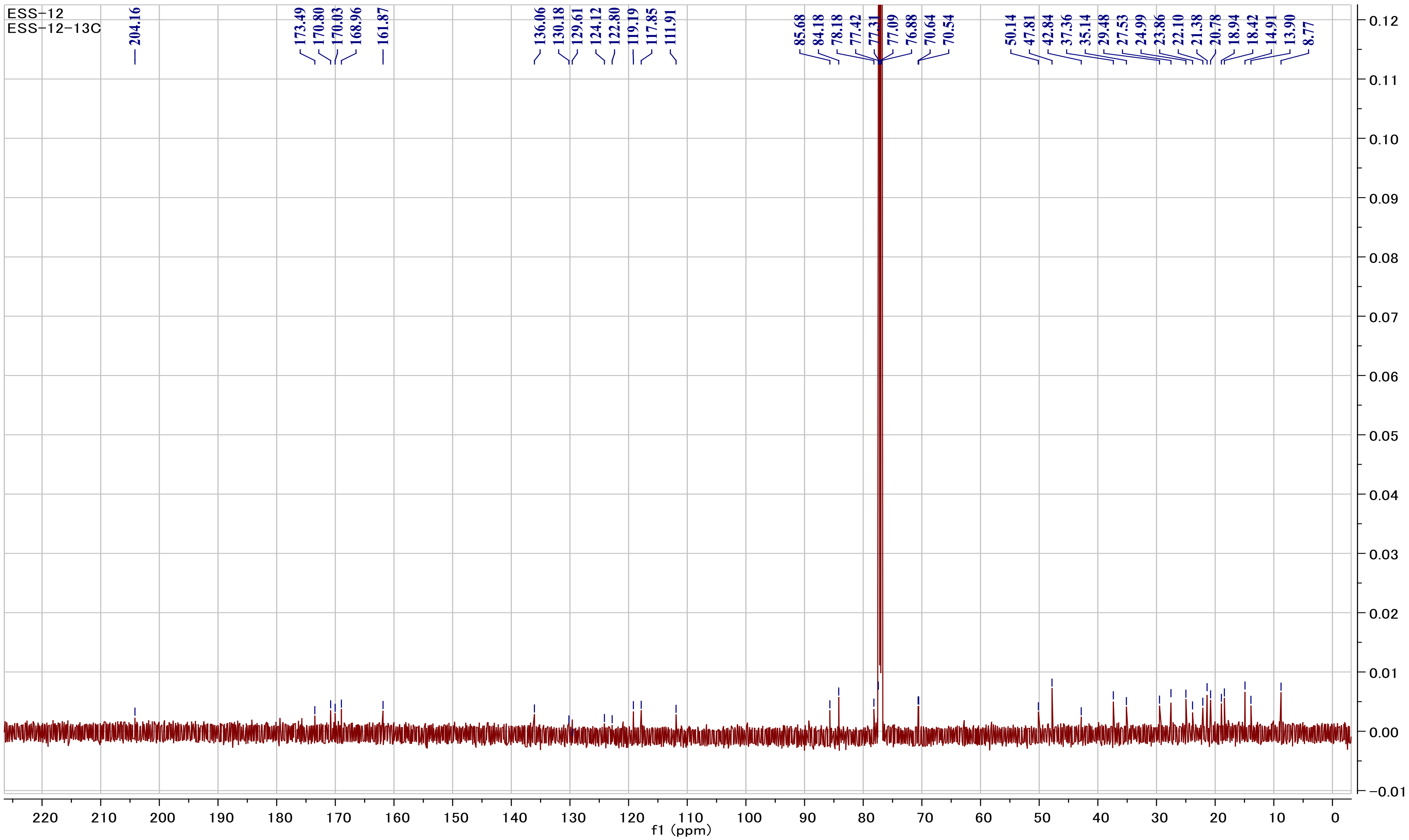

S5: ^{13}C NMR (600 Hz, $CDCl_3$) of **1**

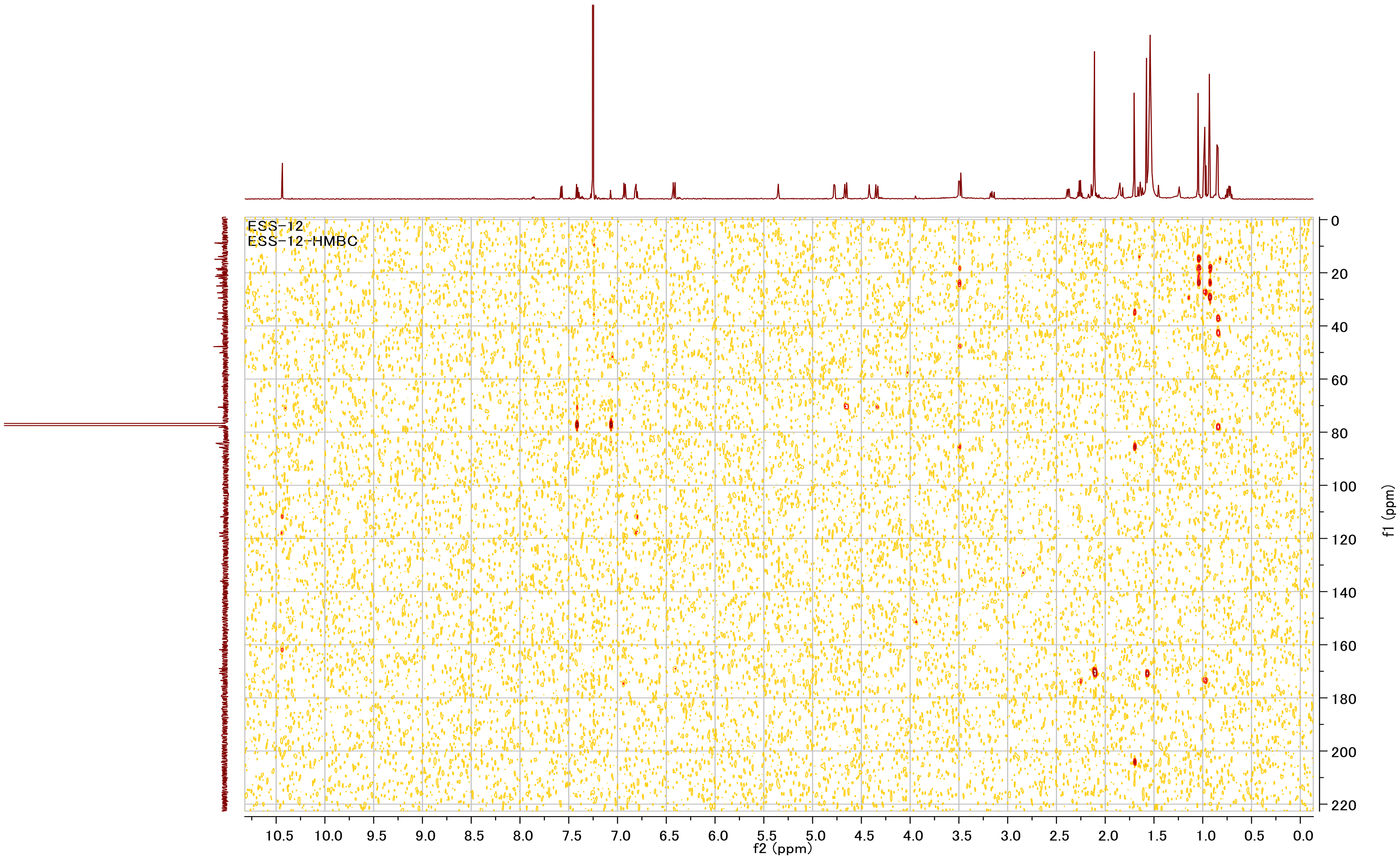

S6: HMQC ($CDCl_3$) of **1**

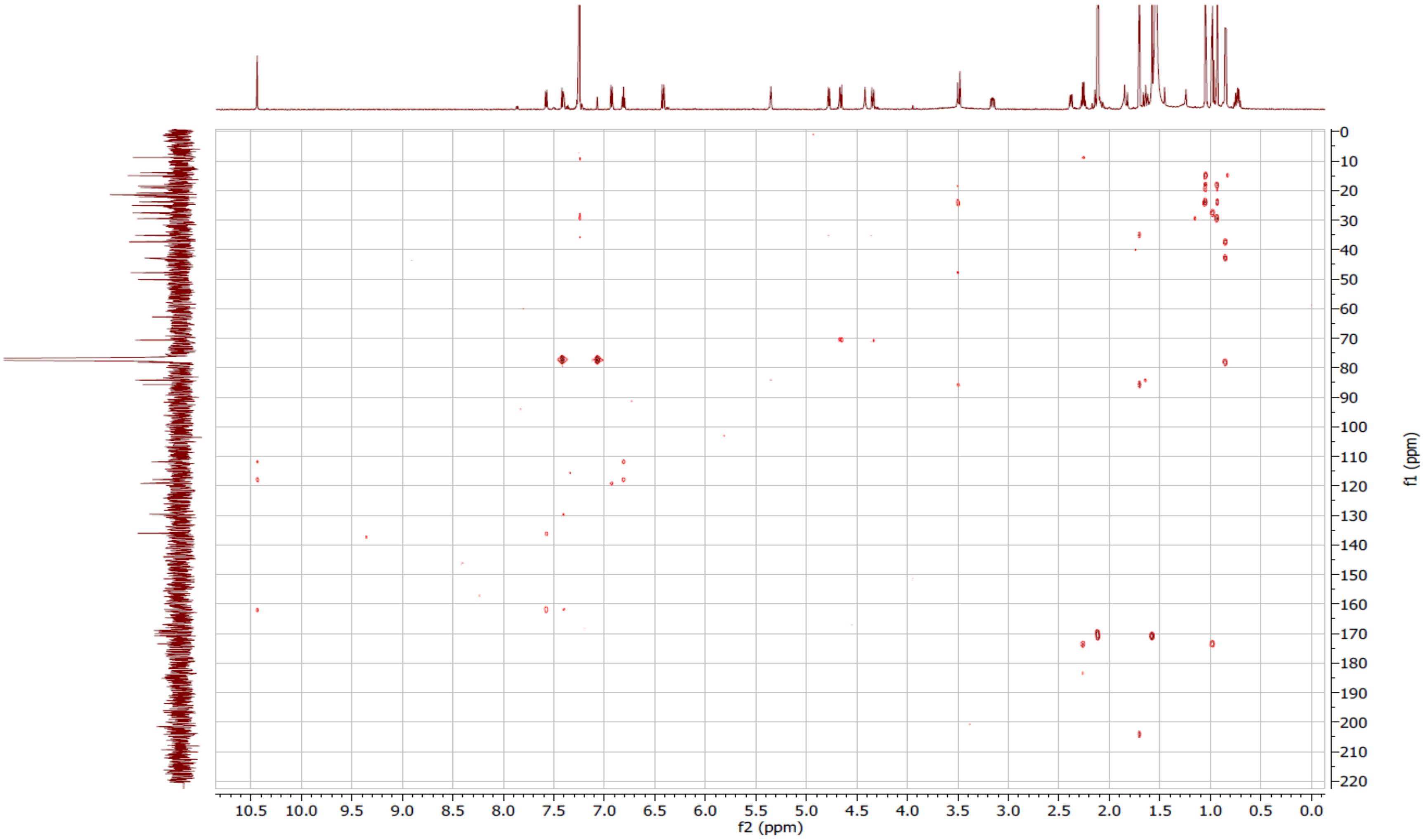

S7: HMBC ($CDCl_3$) of **1**

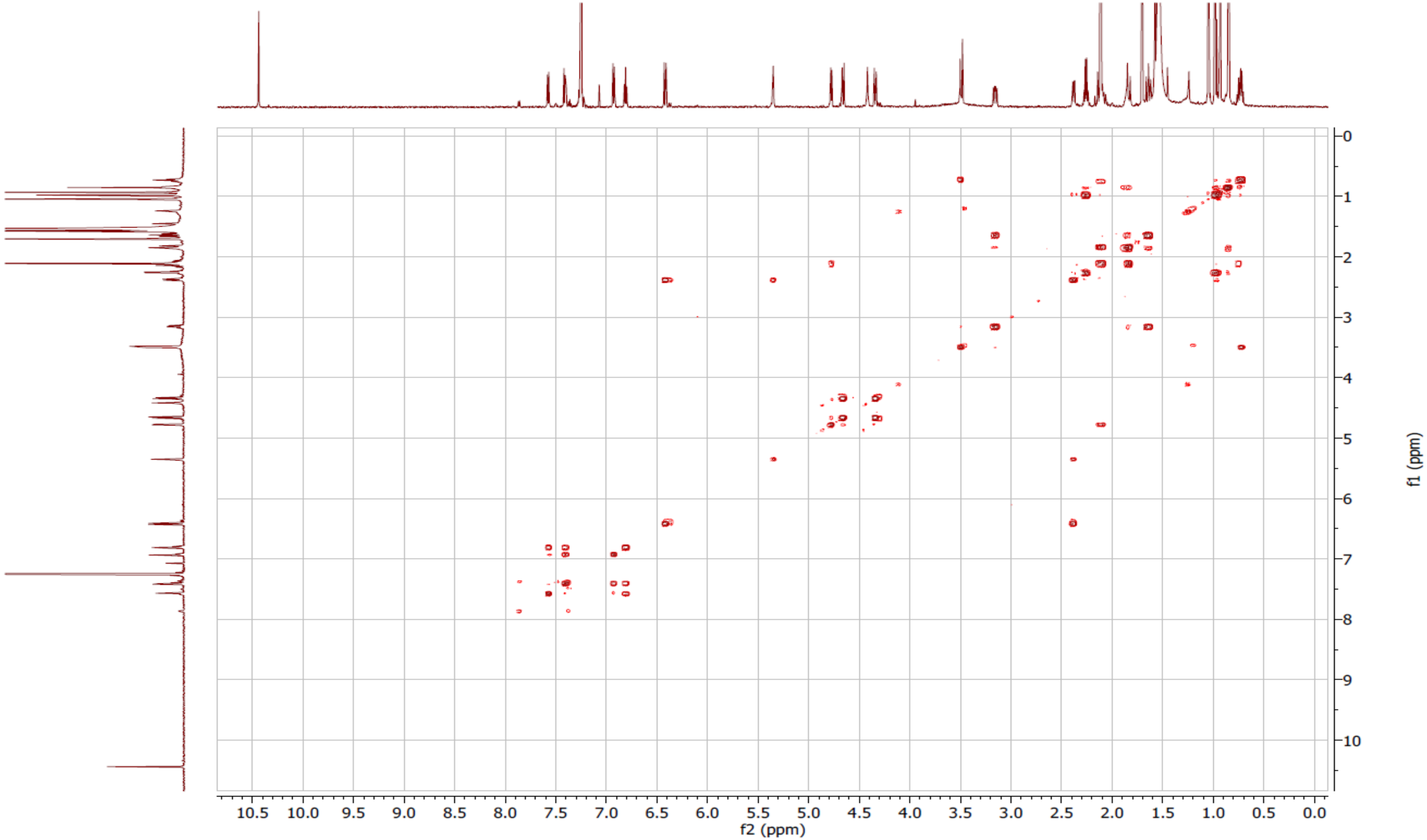

S8: ^{1}H ^{1}H COSY ($CDCl_3$) of **1**

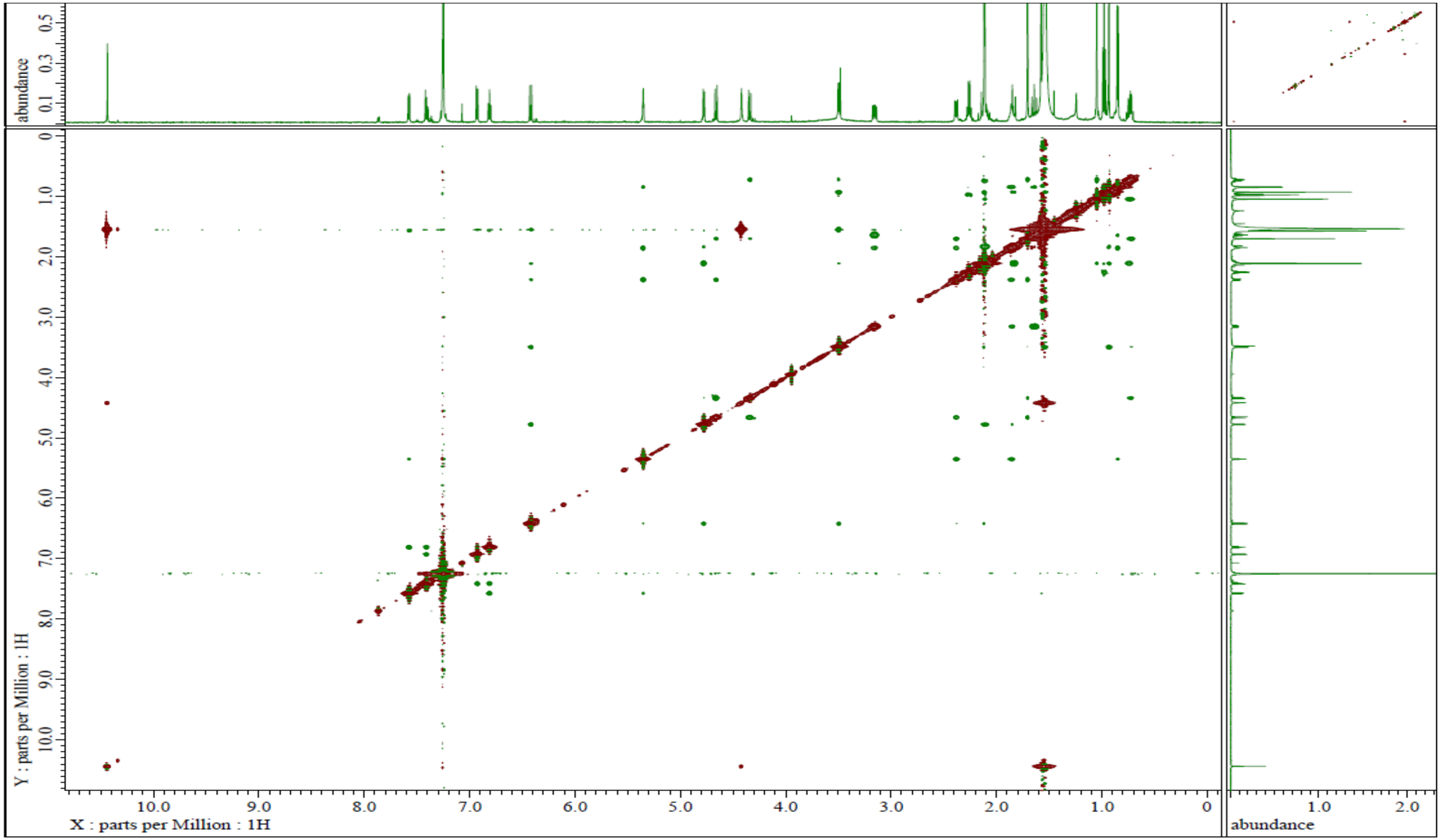

S9: NOESY ($CDCl_3$) of **1**

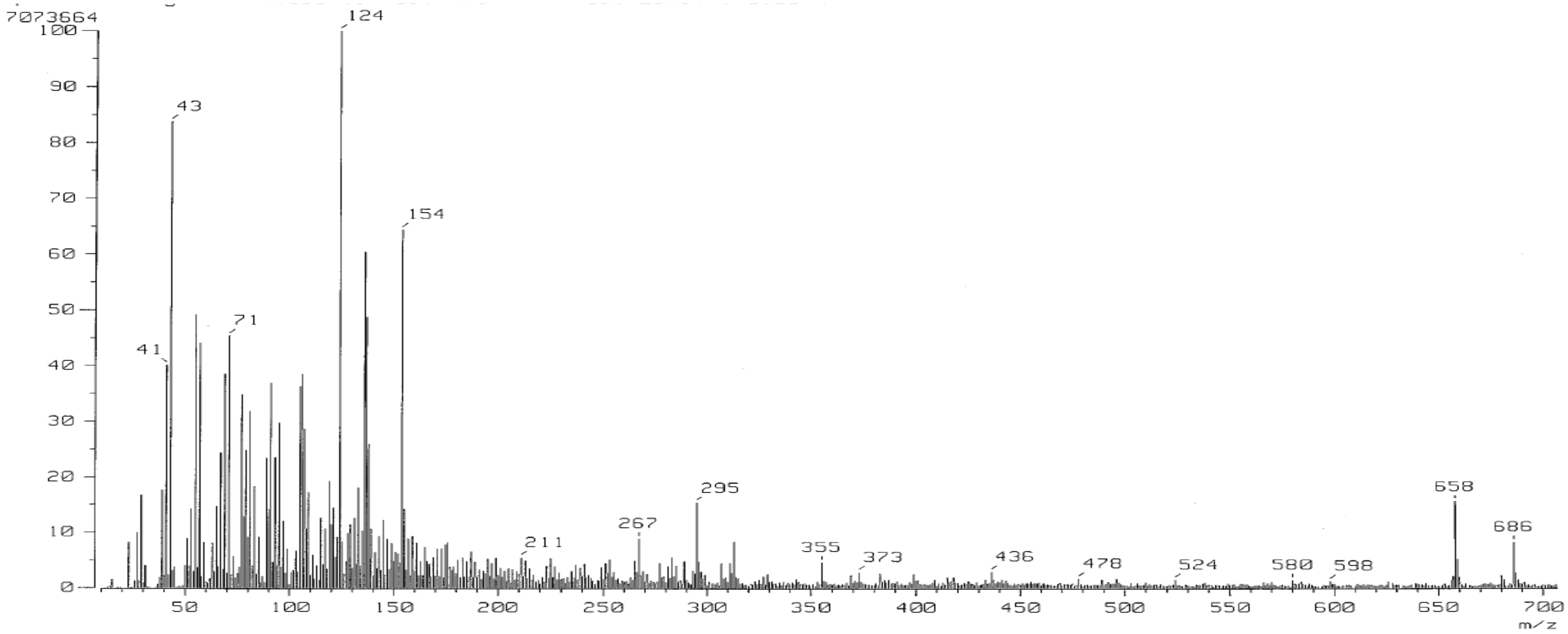

S10: FAB-MS of **2**

Date : 23-Jul-2018 16:12
Instrument: MS700D
Sample: ESS-23
Note: MStation
Inlet: Direct   Ion Mode: FAB+
RT: 3.24 min   Scan#: 19
Elements: C 150/0, H 250/0, O 50/0
Mass Tolerance: 5mmu
Unsaturation (U.S.): 0.0 – 15.0

	Observed m/z	Int%	Err. [ppm I mmu]	U.S	Composition
1	685.3089	100.00	–1.1 / –0.6	13.5	C36 H47 N O12

S11: HR-FAB-MS of **2**

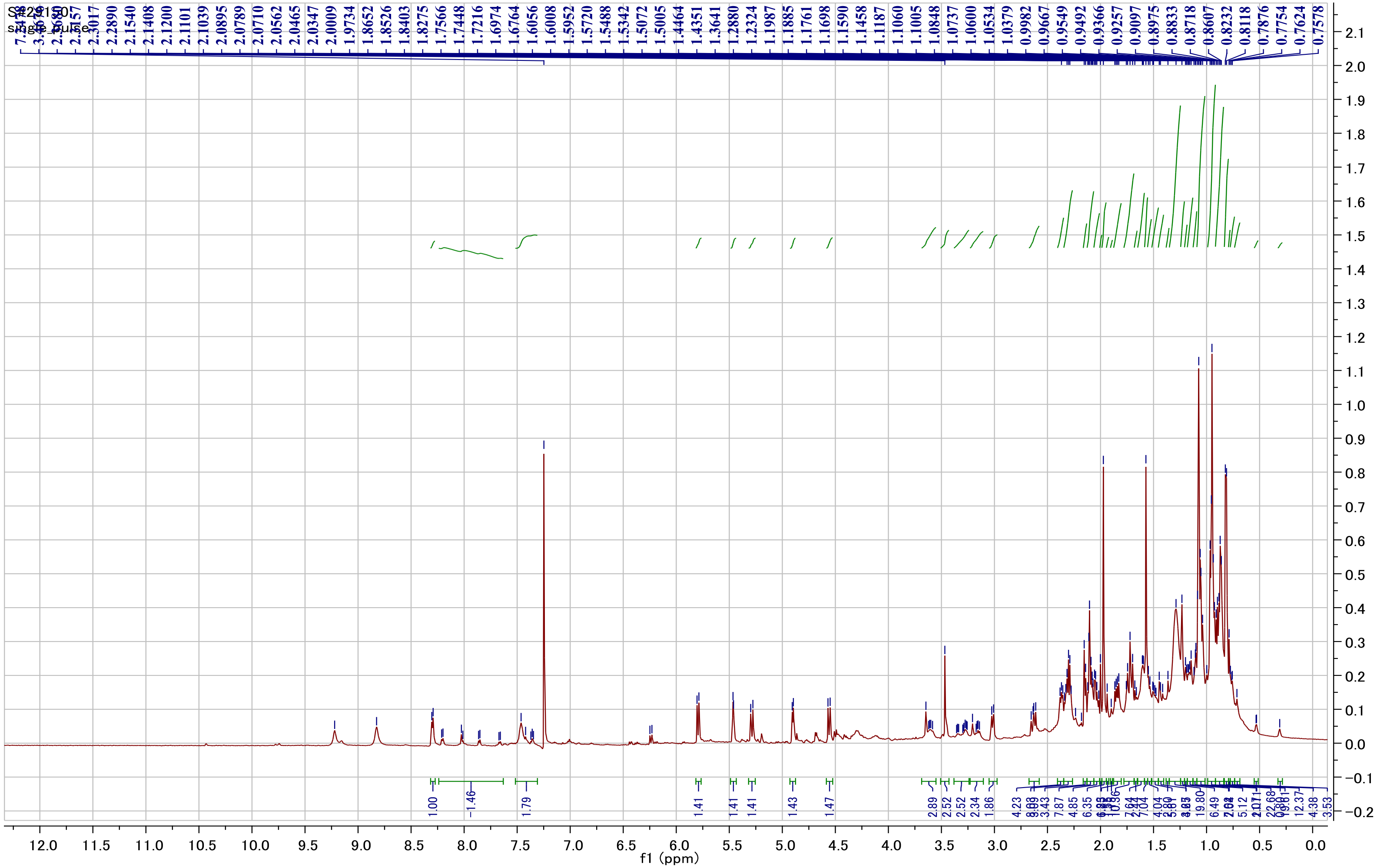

S12: ^{1}H NMR (600 Hz, $CDCl_3$) of **2**

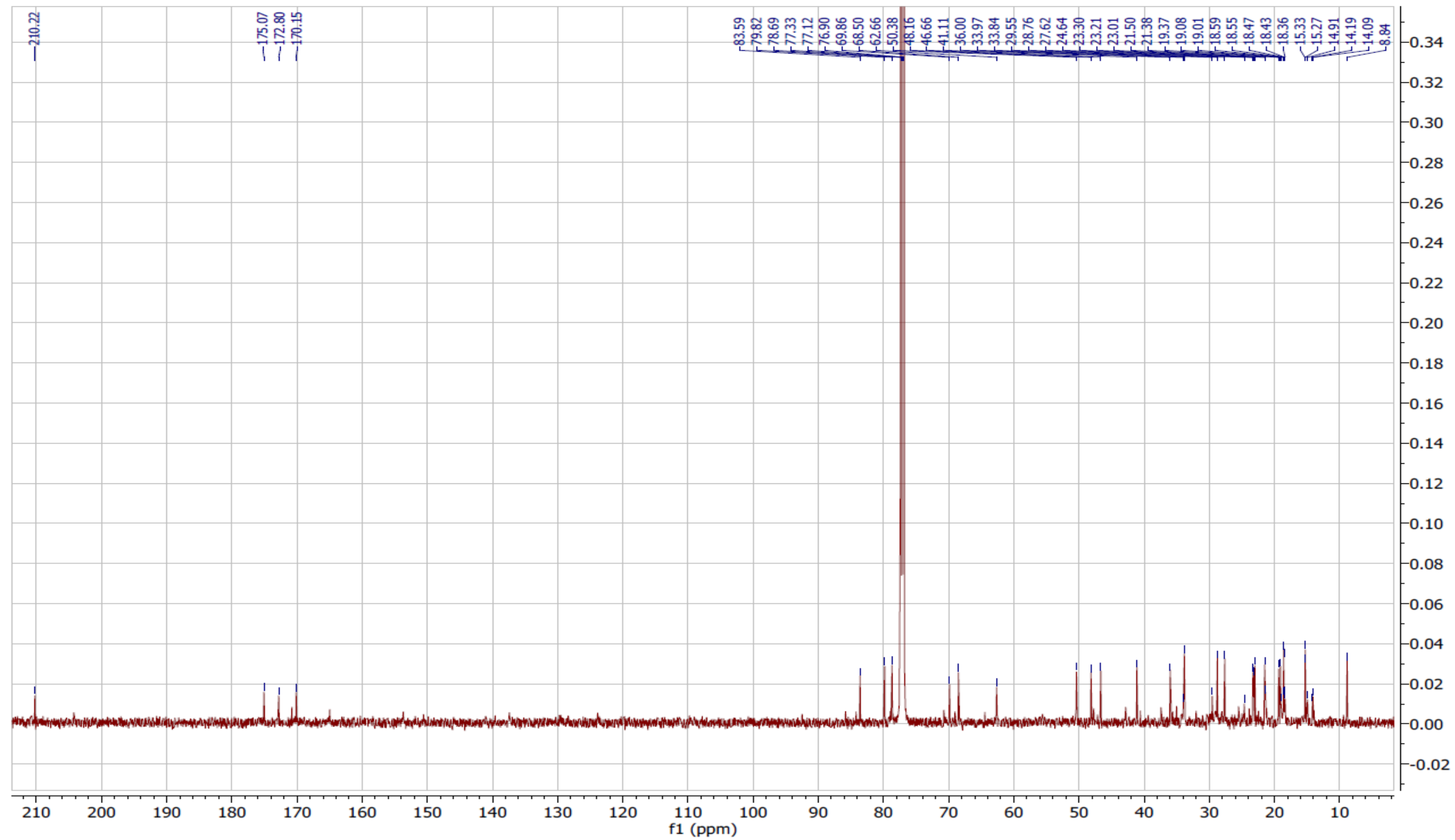

S13: ^{13}C NMR (600 Hz, $CDCl_3$) of **2**

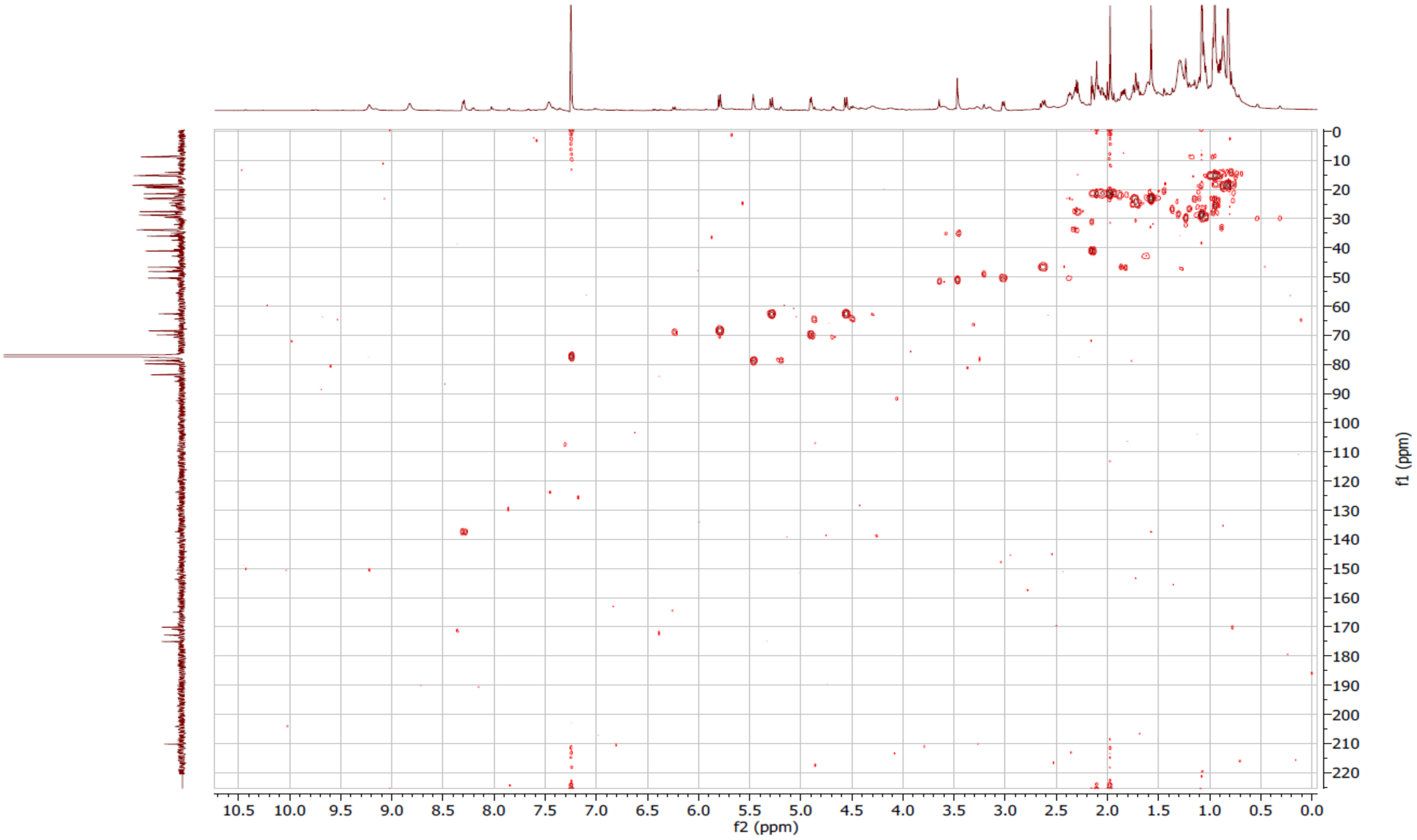

S14: HMQC ($CDCl_3$) of **2**

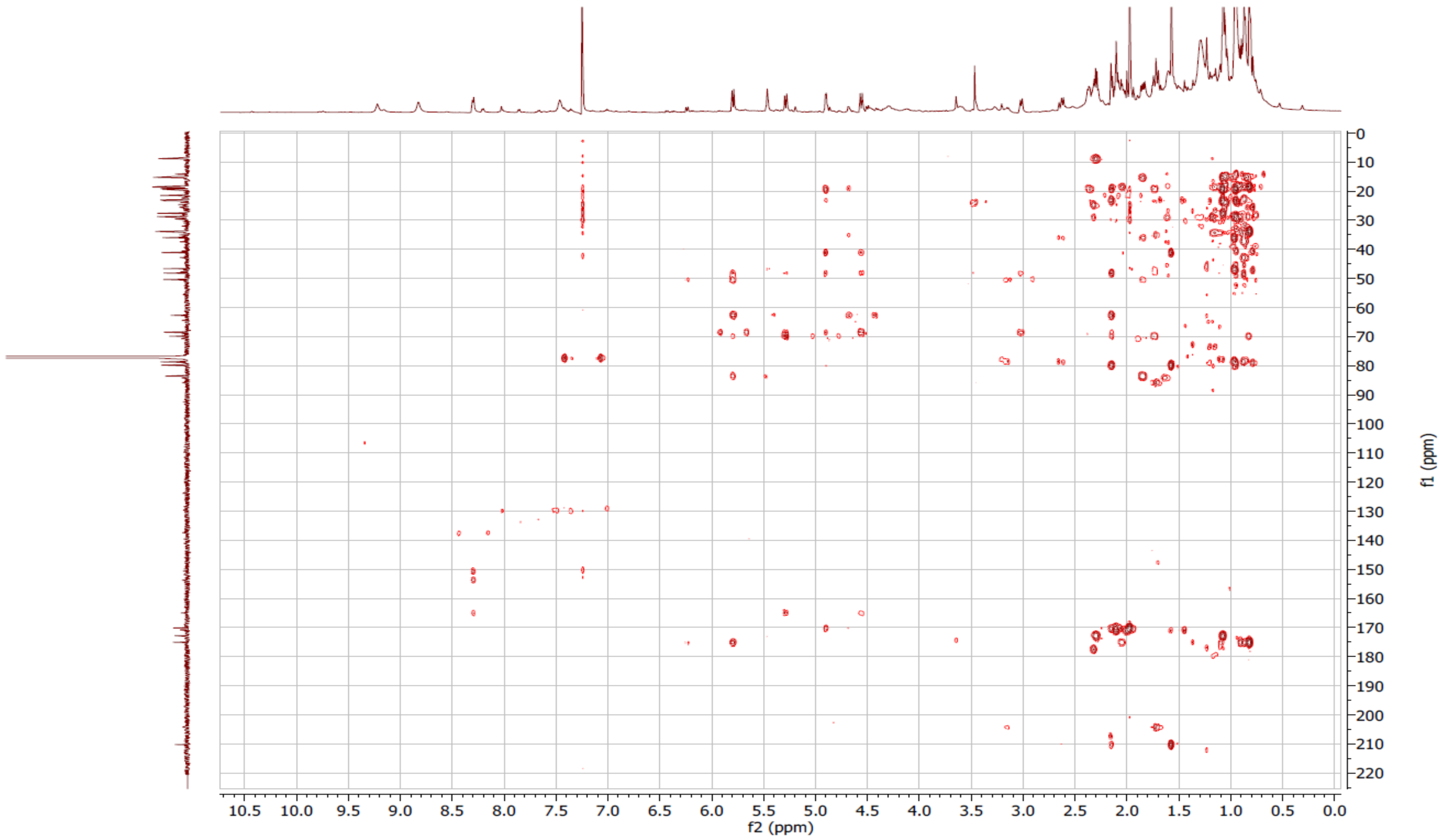

S15: HMBC ($CDCl_3$) of **2**

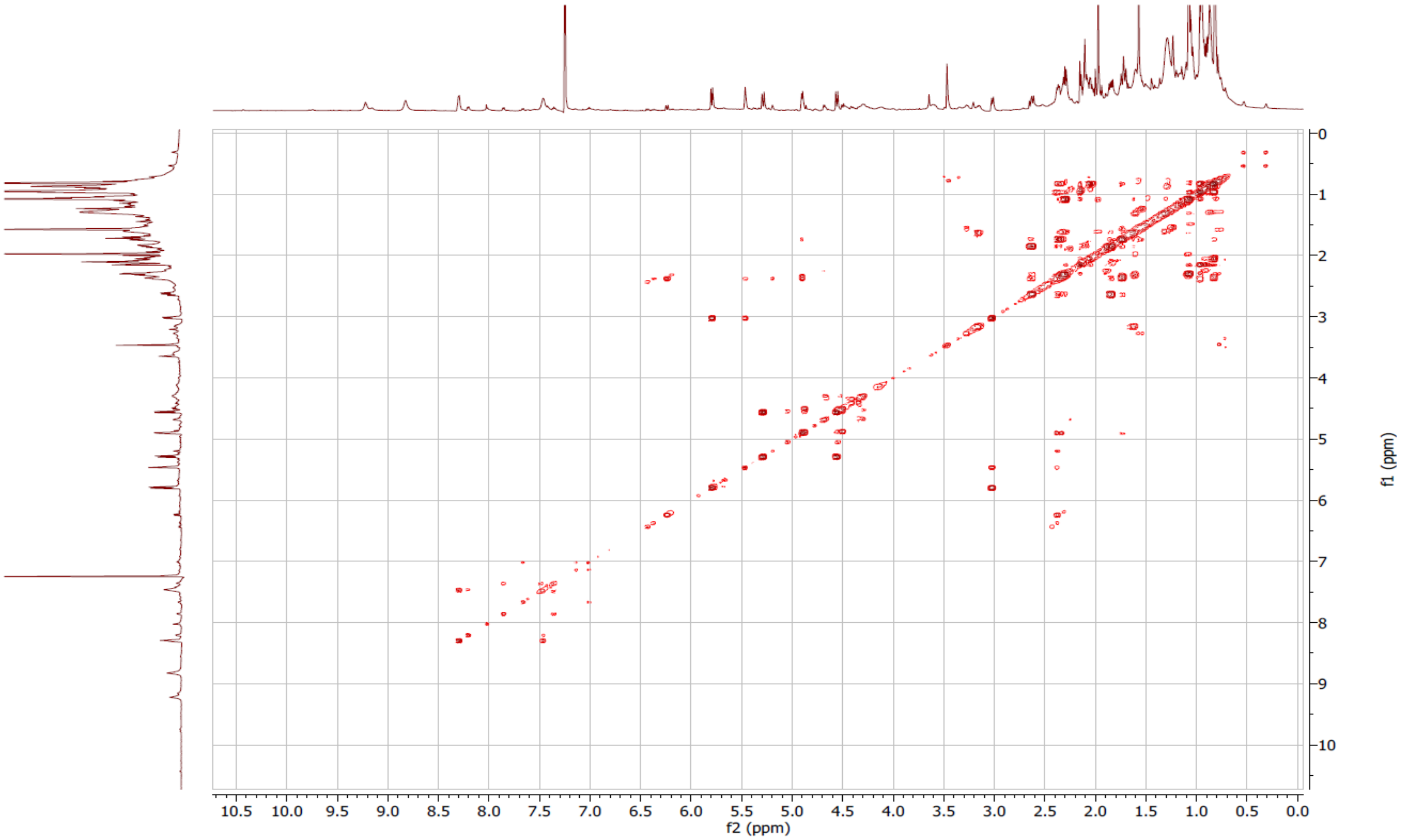

S16: ^1H ^1H COSY ($CDCl_3$) of **2**

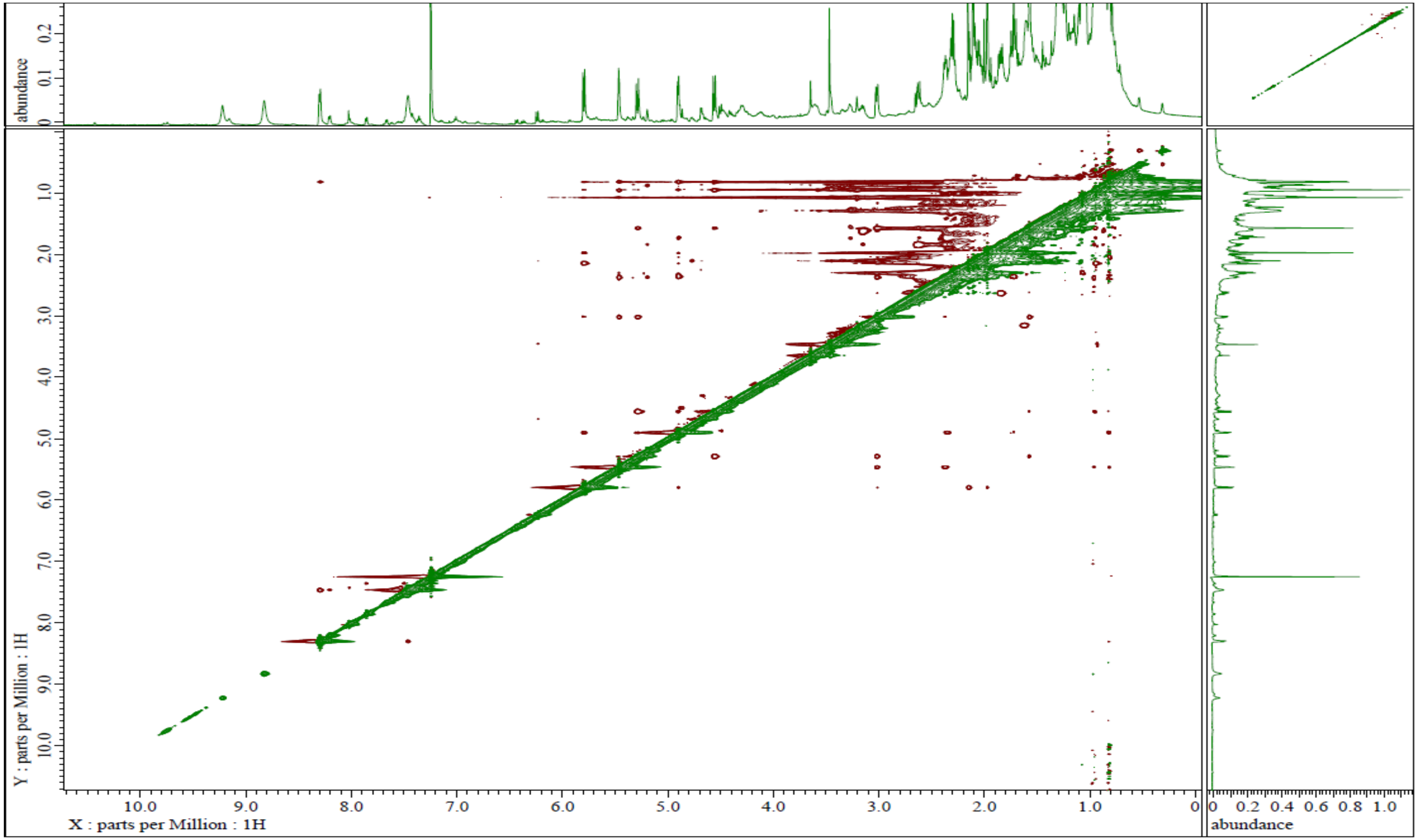

S17: NOESY ($CDCl_3$) of **2**

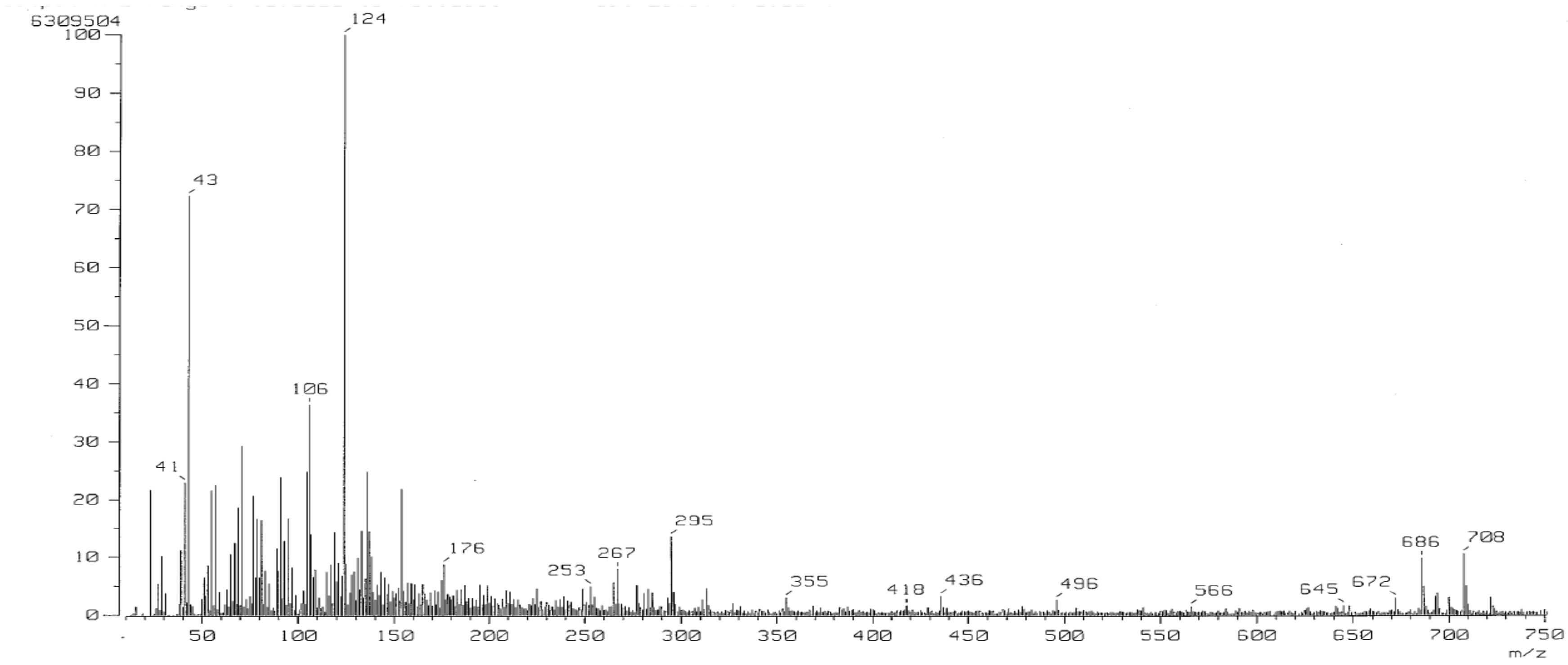

S18: FAB-MS of **3**

Date : 28-Jul-2018 17:31
Instrument: MS700D
Sample: ESS-21
Note: MStation
Inlet: Direct Ion Mode: FAB+
RT: 3.76 min Scan#: 19
Elements: C 150/0, H 250/0, O 50/0
Mass Tolerance: 5mmu
Unsaturation (U.S.): 0.0 – 15.0

	Observed m/z	Int %	Err. [ppm / mmu]	U.S.	Composition
1	708.3090	100.00	–1.1 / –0.6	13.5	C36 H47 N O12 Na

S19: FAB-MS of **3**

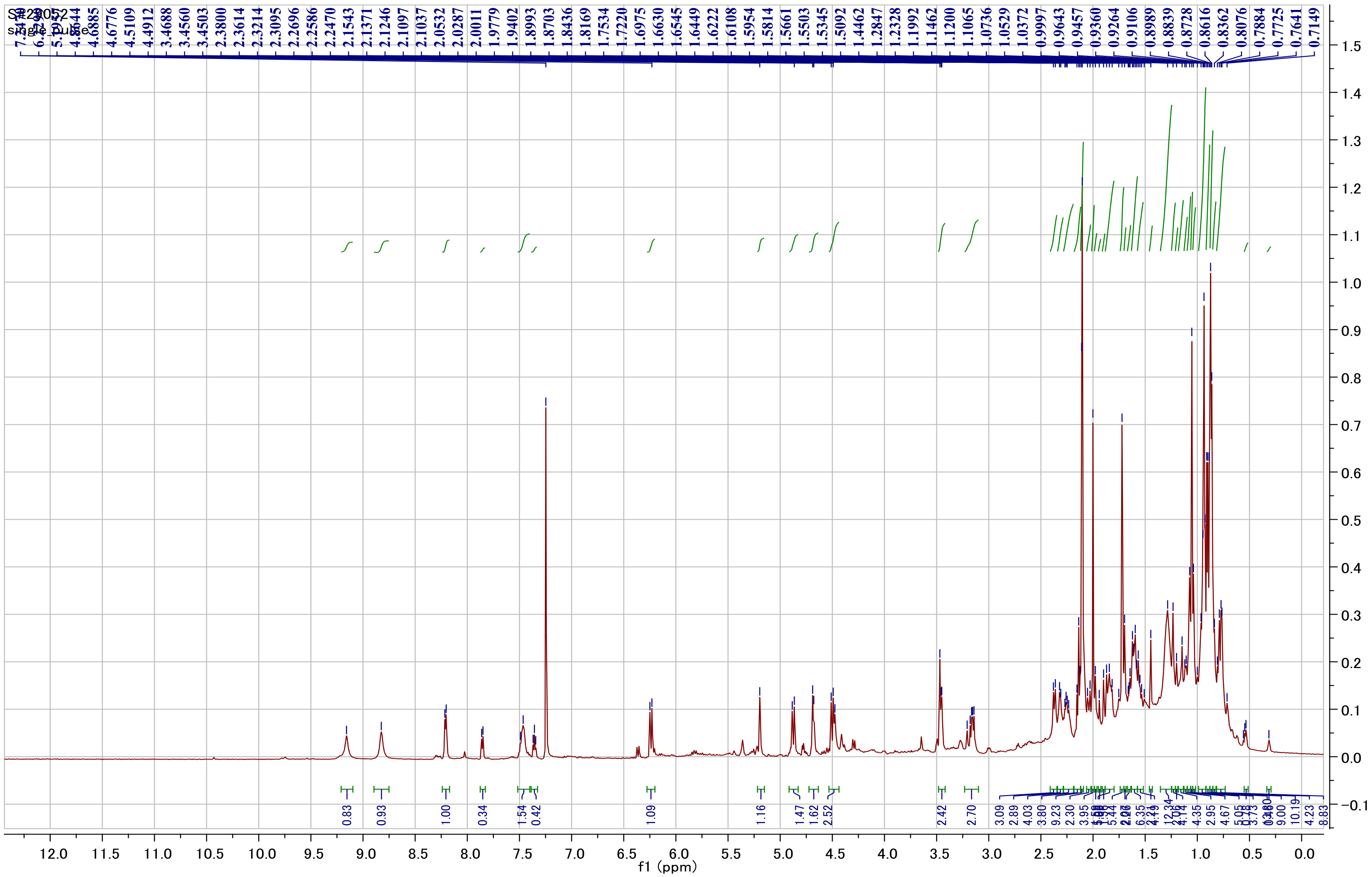

S20: ^{1}H NMR (600 Hz, $CDCl_3$) of **3**

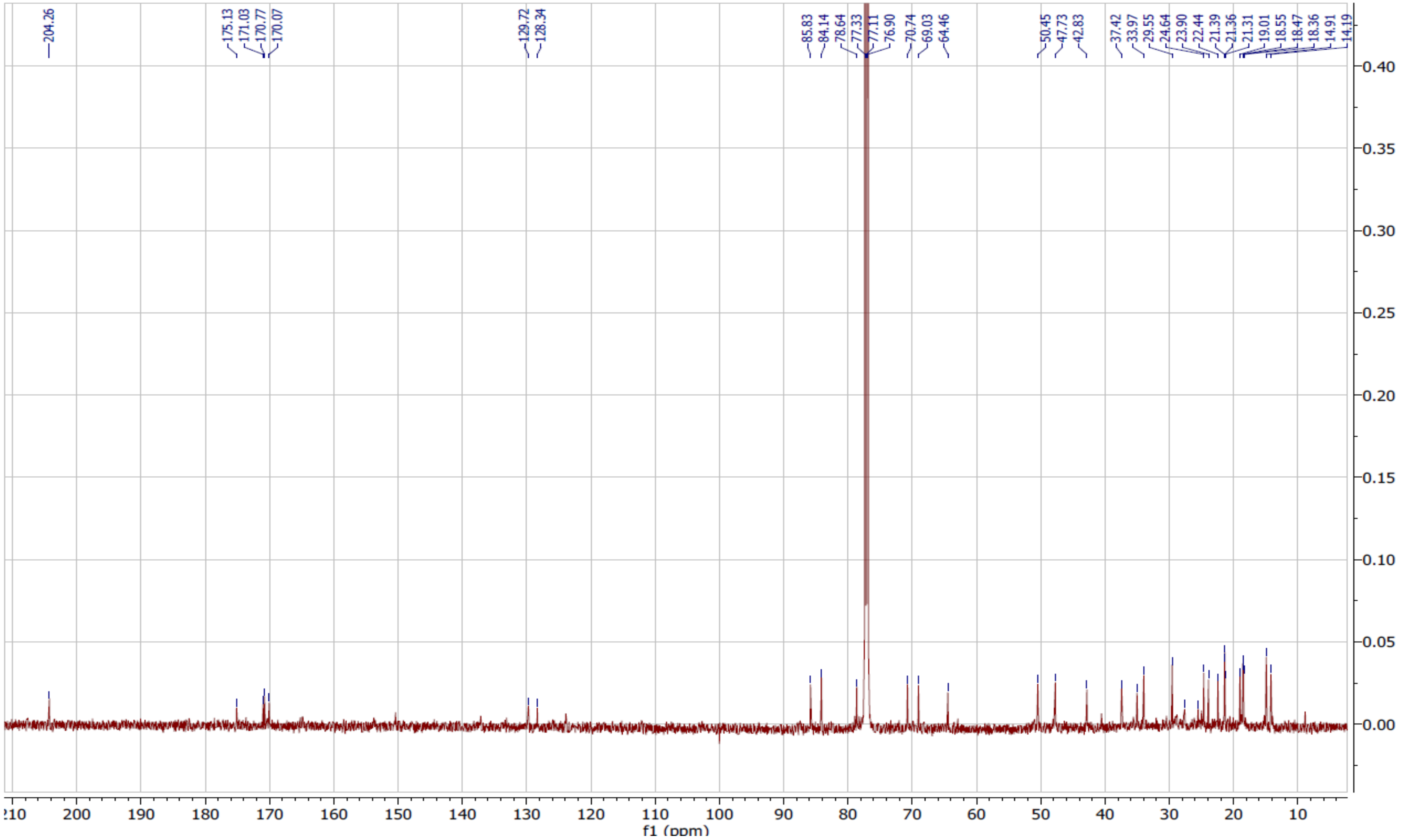

S21: ^{13}C NMR (600 Hz, $CDCl_3$) of **3**

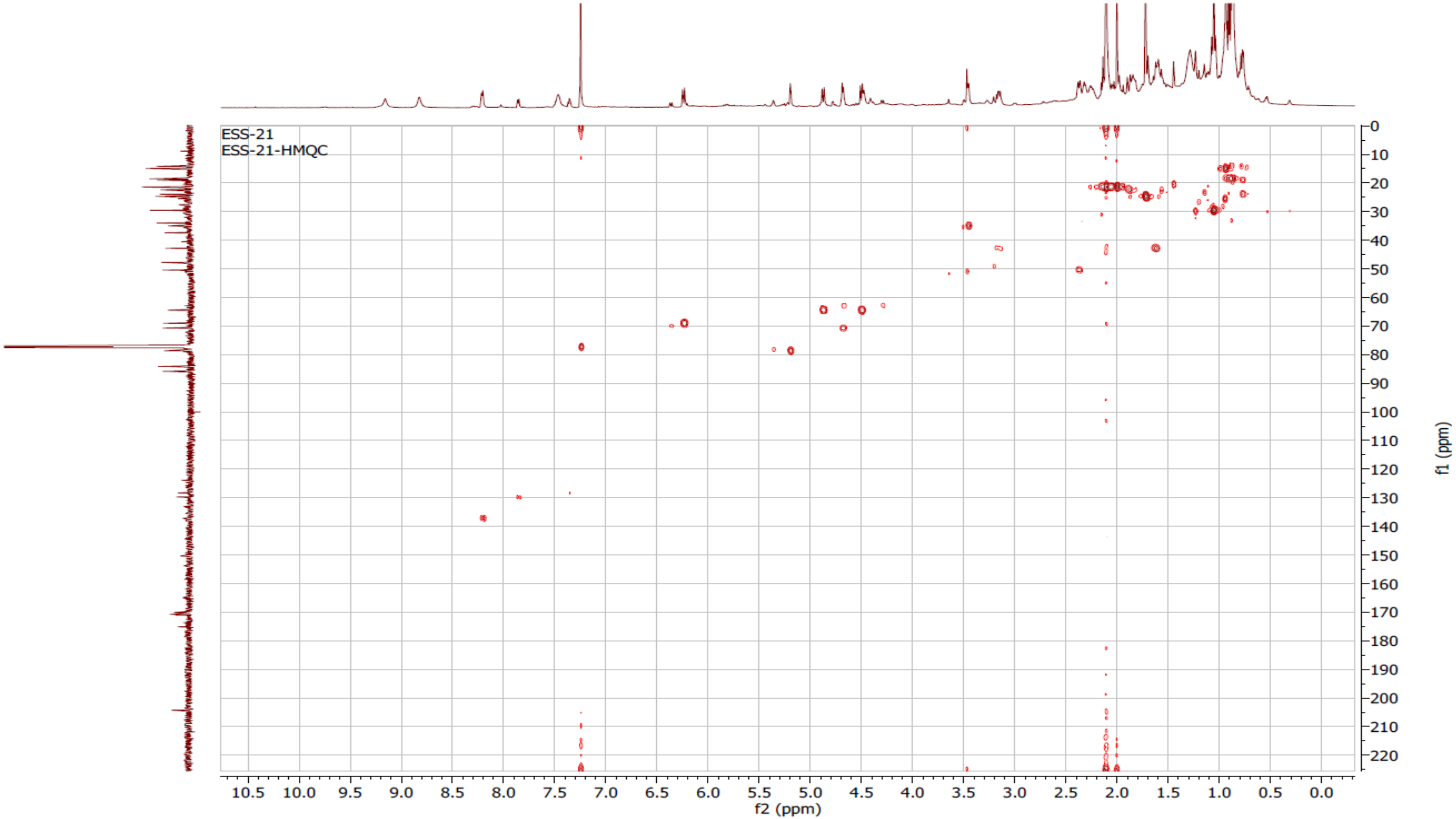

S22: HMQC of **3**

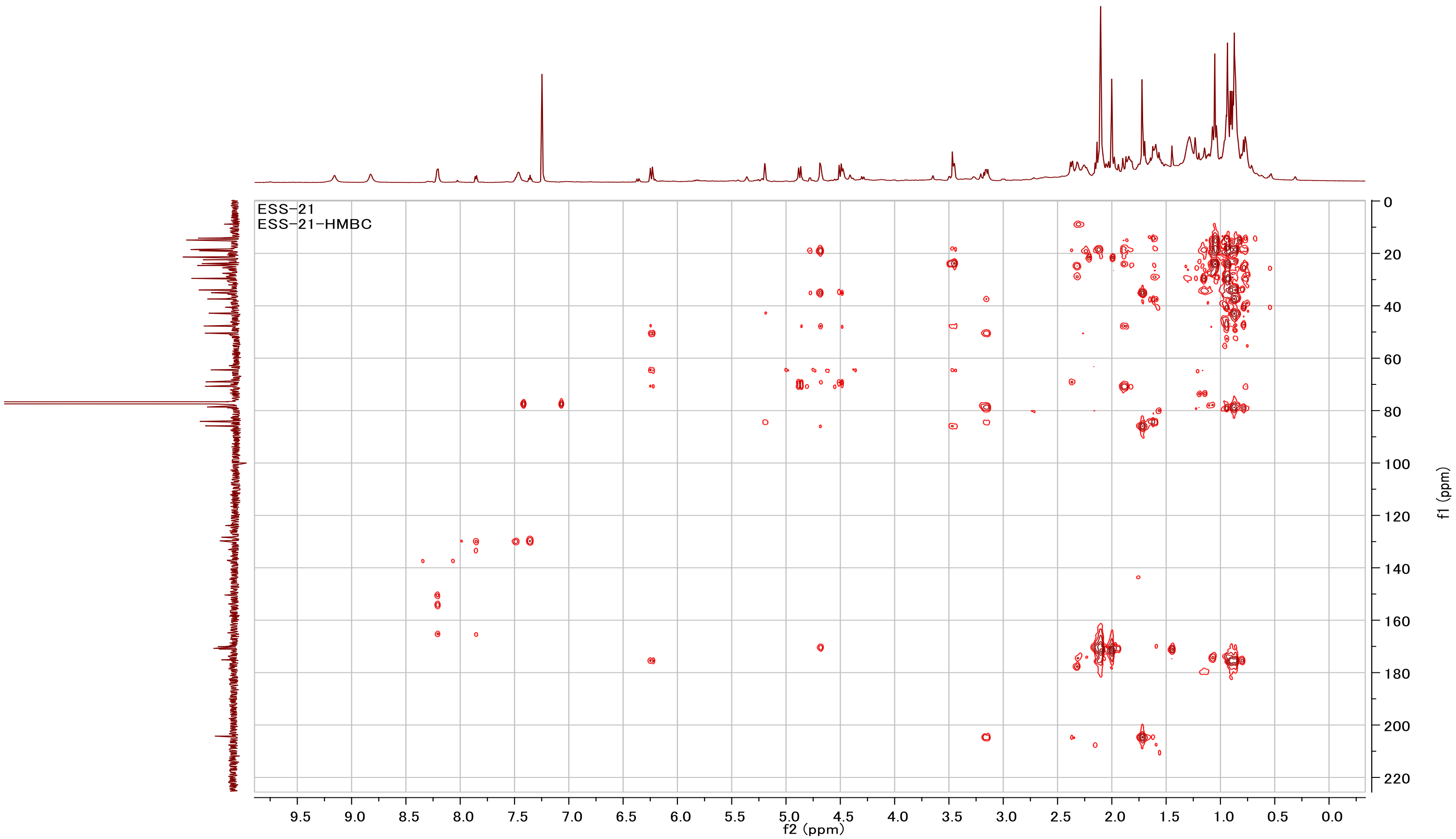

S23: HMBC of **3**

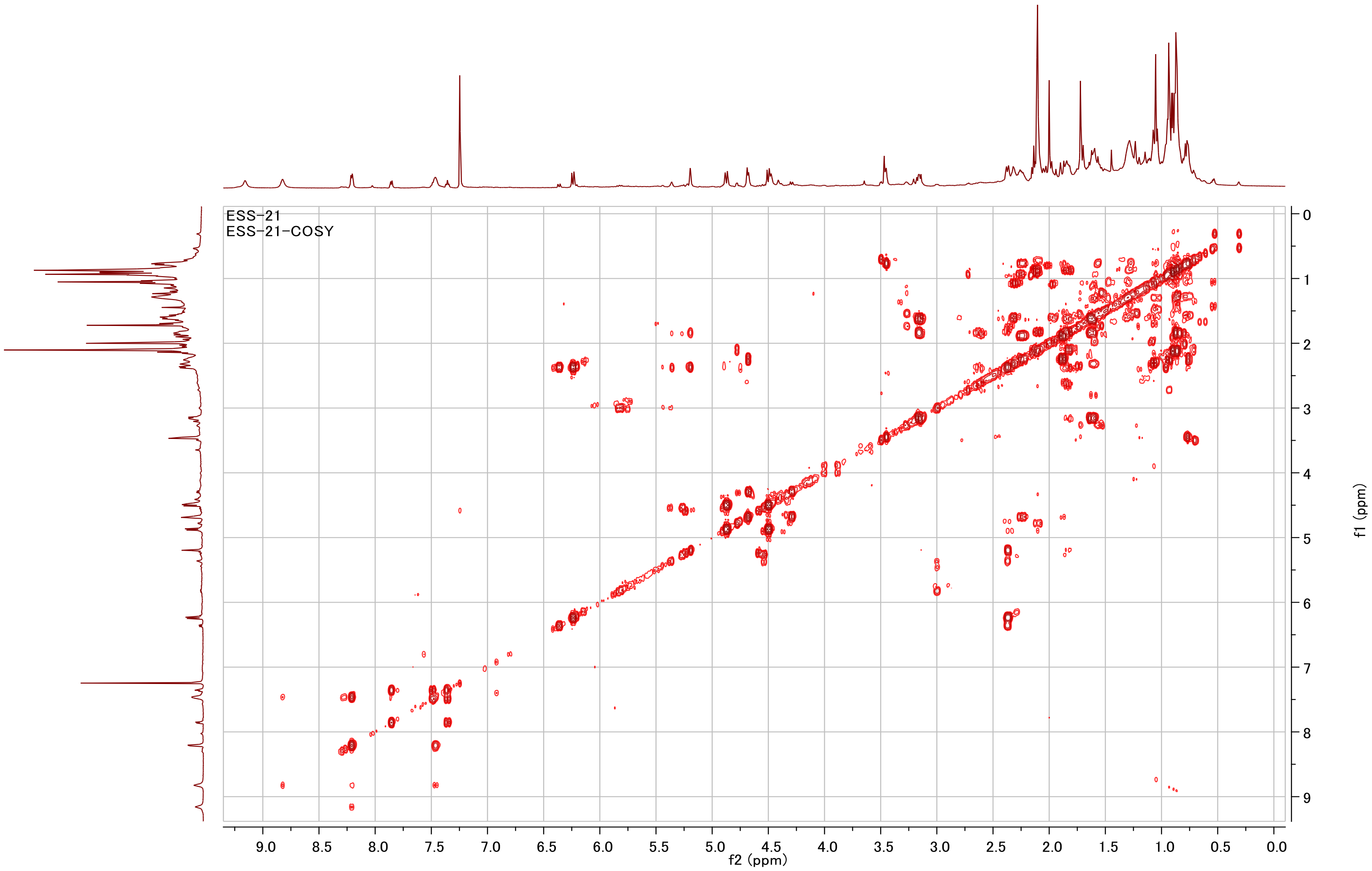

S24: ^{1}H-^{1}H COSY of **3**

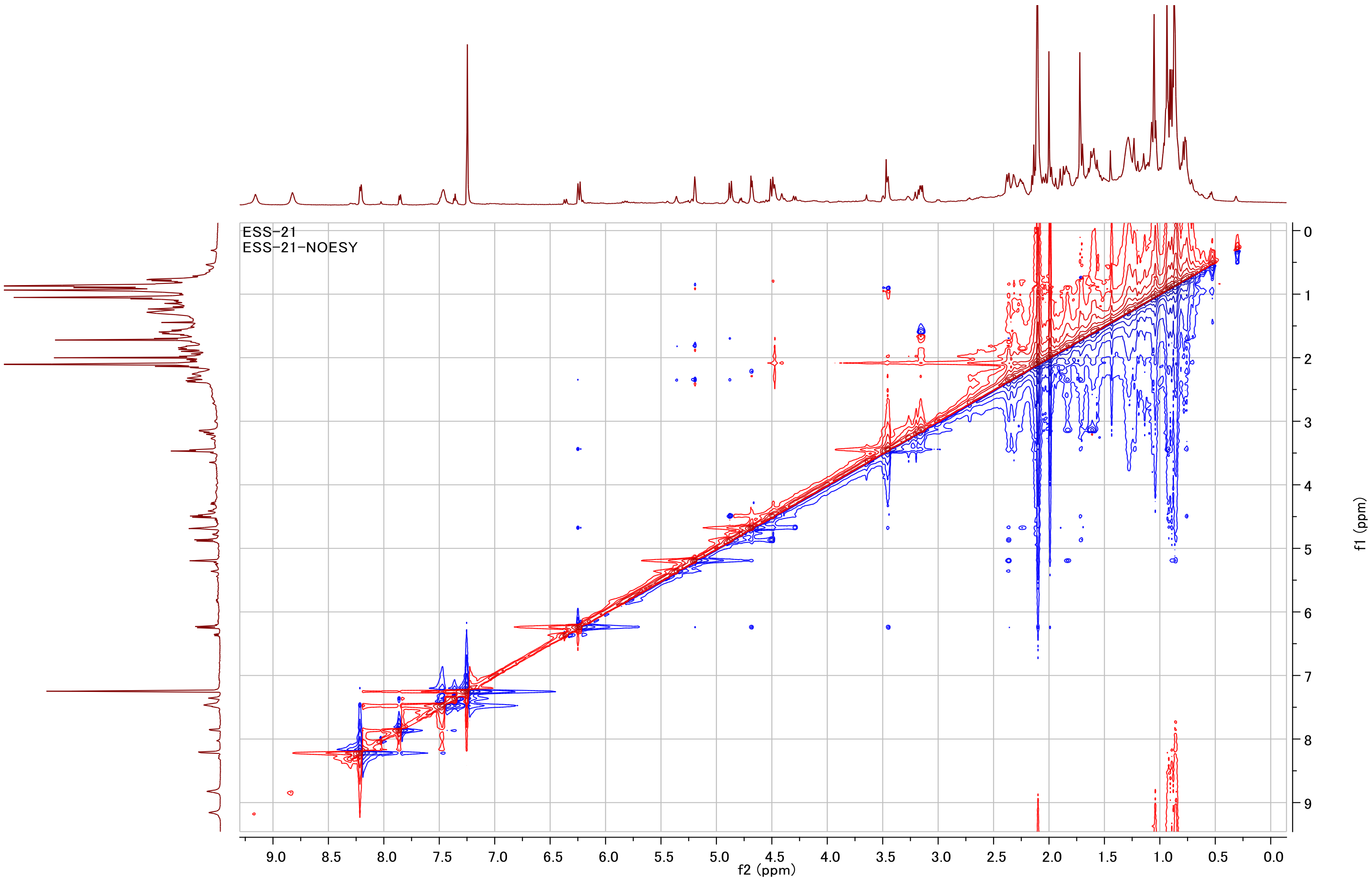

S25: NOESY of **3**
